# Molecular signature for senile and complicated cataracts derived from analysis of sumoylation enzymes and their substrates in human cataract lenses

**DOI:** 10.1111/acel.13222

**Published:** 2020-08-22

**Authors:** Fang‐Yuan Liu, Jia‐Ling Fu, Ling Wang, Qian Nie, Zhongwen Luo, Min Hou, Yuan Yang, Xiao‐Dong Gong, Yan Wang, Yuan Xiao, Jiawen Xiang, Xuebin Hu, Lan Zhang, Mingxing Wu, Weirong Chen, Bing Cheng, Lixia Luo, Xinyu Zhang, Xialin Liu, Danying Zheng, Shengsong Huang, Yizhi Liu, David Wan‐Cheng Li

**Affiliations:** ^1^ State Key Laboratory of Ophthalmology Zhongshan Ophthalmic Center Sun Yat‐Sen University Guangzhou China

**Keywords:** aging, apoptosis, cataract, de‐sumoylation enzymes (SENPs), Pax6, SUMO1, SUMO2/3, sumoylation ligases

## Abstract

Sumoylation is one of the key regulatory mechanisms in eukaryotes. Our previous studies reveal that sumoylation plays indispensable roles during lens differentiation (Yan et al. 2010. *Proc Natl Acad Sci USA*. 107:21034–21039; Gong et al. 2014. *Proc Natl Acad Sci USA*. 111:5574–5579). Whether sumoylation is implicated in cataractogenesis, a disease largely derived from aging, remains elusive. In the present study, we have examined the changing patterns of the sumoylation ligases and de‐sumoylation enzymes (SENPs) and their substrates including Pax6 and other proteins in cataractous lenses of different age groups from 50 to 90 years old. It is found that compared with normal lenses, sumoylation ligases 1 and 3, de‐sumoylation enzymes SENP3/7/8, and p46 Pax6 are clearly increased. In contrast, Ubc9 is significantly decreased. Among different cataract patients from 50s to 70s, male patients express more sumoylation enzymes and p46 Pax6. Ubc9 and SENP6 display age‐dependent increase. The p46 Pax6 displays age‐dependent decrease in normal lens, remains relatively stable in senile cataracts but becomes di‐sumoylated in complicated cataracts. In contrast, sumoylation of p32 Pax6 is observed in senile cataracts and increases its stability. Treatment of rat lenses with oxidative stress increases Pax6 expression without sumoylation but promotes apoptosis. Thus, our results show that the changing patterns in Ubc9, SENP6, and Pax6 levels can act as molecular markers for senile cataract and the di‐sumoylated p46 Pax6 for complicated cataract. Together, our results reveal the presence of molecular signature for both senile and complicated cataracts. Moreover, our study indicates that sumoylation is implicated in control of aging and cataractogenesis.

## Graphical abstract

The molecular signatures for both senile cataract and complicated cataract. Upregulation of sumoylation ligases Ubc9 and PIAS1, and de‐sumoylation enzyme SENP6, and SUMO1 conjugation of p32 Pax6 are molecular markers for senile cataract. Di‐sumoylation of p46 Pax6 is the molecular marker for complicated cataract.

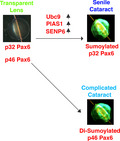

**Abbreviations**
AD: Alzheimer's disease
APP: amyloid precursor protein
AWB: automated Western blot
GO: glucose oxidase
HD: homeodomain
OS: oxidative stress
PAGE: polyacrylamide gel electrophoresis
PBS: phosphate‐buffered saline
PD: paired domain
SDS: sodium dodecyl sulfate
SENPs: cysteine proteases of the sentrin‐specific protease family
SUMO1: small ubiquitin‐like molecule 1
SUMO2/3: small ubiquitin‐like molecule 2/3
TBS: Tris‐buffered saline
TBS‐T: Tris‐buffered saline with Tween‐20
αTN4‐1: mouse lens epithelial cell line

## 1 INTRODUCTION

Sumoylation is now established as one of the key regulatory mechanisms in eukaryotic cells, regulating functions of more than 3,600 different proteins implicated in control of many physiological processes (Flotho & Melchior, 2013; Gareau & Lima, 2010; Geiss‐Friedlander & Melchior, 2007; Hendriks & Vertegaal, 2016; Johnson, 2004). Moreover, it also acts as a molecular mechanism mediating global changes at the cellular and organism levels when stress conditions such as heat shock or oxidative stress occur (Flotho & Melchior, 2013).

Sumoylation is an enzyme‐catalyzed reversible biochemical reaction in which the SUMO proteins (SUMO1, SUMO2, or SUMO3) are conjugated to the target protein through specific conserved sumoylation site (Flotho & Melchior, 2013; Gareau & Lima, 2010; Geiss‐Friedlander & Melchior, 2007; Hendriks & Vertegaal, 2016; Johnson, 2004). The typical motif of sumoylation in the target protein is ψKxE (ψ, a large hydrophobic residue; x, any amino acid). Three types of enzymes are involved in accomplishing the sumoylation reaction with the consumption of ATP as energy supply. These enzymes are the E1‐activating enzyme consisting of two subunits, AOS1 and UBA2; the solely E2‐conjugating enzyme, Ubc9; and multiple E3 ligases including RanPB2, PIAS1, and many others (Flotho & Melchior, 2013; Gareau & Lima, 2010; Geiss‐Friedlander & Melchior, 2007; Hendriks & Vertegaal, 2016; Johnson, 2004). On the other hand, sumoylated proteins are de‐sumoylated by several SUMO isopeptidases named SENPs (SENP1, SENP2, SENP3, SENP5, SENP6, SENP7, and SENP8). The SENP enzymes are also responsible for the maturation of the precursor SUMO proteins (Hickey, Wilson, & Hochstrasser, 2012; Kunz, Piller, & Müller, 2018).

In the vertebrate eye, sumoylation has been shown to play very important roles in regulating differentiation of various ocular tissues (Gong et al., 2014; Onishi, Peng, Chen & Blackshaw, 2010; Onishi et al., 2009; Roger, Nellissery, Kim & Swaroop, 2010; Yan et al., 2010). During retina differentiation, the SUMO E3 ligase and transcription cofactor Pias3‐dependent sumoylation of photoreceptor‐specific transcription factors (TFs) have been identified to regulate distinct molecular targets, thereby controlling rod and cone photoreceptor subtype specification (Onishi et al., 2009, 2010; Roger et al., 2010). In the ocular lens, we have previously shown that SUMO1‐mediated conjugation of p32 Pax6 is essential to activate its function (Yan et al., 2010). The activated Pax6 is implicated in modulating differentiation of both retina and lens. More recently, we demonstrated that different SUMOs can act on the same transcription factor to display contrast functions in regulating lens differentiation (Gong et al., 2014).

Accumulated evidences have shown that sumoylation also plays causal roles in many human diseases such as cardiovascular, neuronal diseases, and cancers (Ballatore, Lee & Trojanowski, 2007; Fatkin et al., 1999; Kim et al., 2006; Kim et al., 2007; Li et al., 2003; Steffan et al., 2004; Sternsdorf et al., 1999; Subramaniam, Sixt, Barrow & Snyder, 2009). In this regard, we have recently revealed that during oxidative stress‐induced age‐related macular degeneration (AMD), de‐sumoylation of p53 is essential to mediate heterochromatin protection of retinal pigmental epithelial cells under the oxidative stress insult (Gong et al., 2018). Whether sumoylation is implicated in lens cataractogenesis (a disease largely derived from aging) remains elusive.

In the present study, by analyzing the protein levels of both ligases and de‐sumoylation enzymes, as well as their substrates in cataract lenses of different age groups without or with other conditions, we have demonstrated that sumoylation is implicated in control of aging and cataractogenesis. Our studies reveal that the protein levels of sumoylation ligases such as Ubc9, de‐sumoylation enzymes such as SENP6, and their substrate, the non‐sumoylated p46 and p32 Pax6, can be used as molecular markers for simple senile cataract. In addition, our results show that the di‐sumoylated p46 Pax6 can be used as a molecular marker for complicated cataract such as diabetic cataract. Together, our results reveal the presence of molecular signature for both senile and complicated cataracts.

## 2 RESULTS

### 2.1 Sumoylation ligases 1 (AOS1 and UBA2), 2 (Ubc9), and 3 (PIAS1 and RanBP2) in cataractous lenses of different age groups

We have previously shown that sumoylation plays an indispensable role in governing lens differentiation (Gong et al., 2014; Yan et al., 2010). Whether sumoylation is implicated in cataractogenesis remains elusive. To obtain the answer to the above question, we examined the protein levels of three different types of ligases in capsular epithelia isolated from three pairs of normal human lenses (two female donors of 45 and 56 years old, and one male donor of 65 years old) and 133 cataract patients with different ages (31 patients of 50–59 years old [Table S1], grouped as 50s; 38 patients of 60–69 years old [Table S2], grouped as 60s, 37 patients of 70–79 years old [Table S3], grouped as 70s, and 27 patients of 80–90 years old [Table S4], grouped as 80s).

**FIGURE 1:**
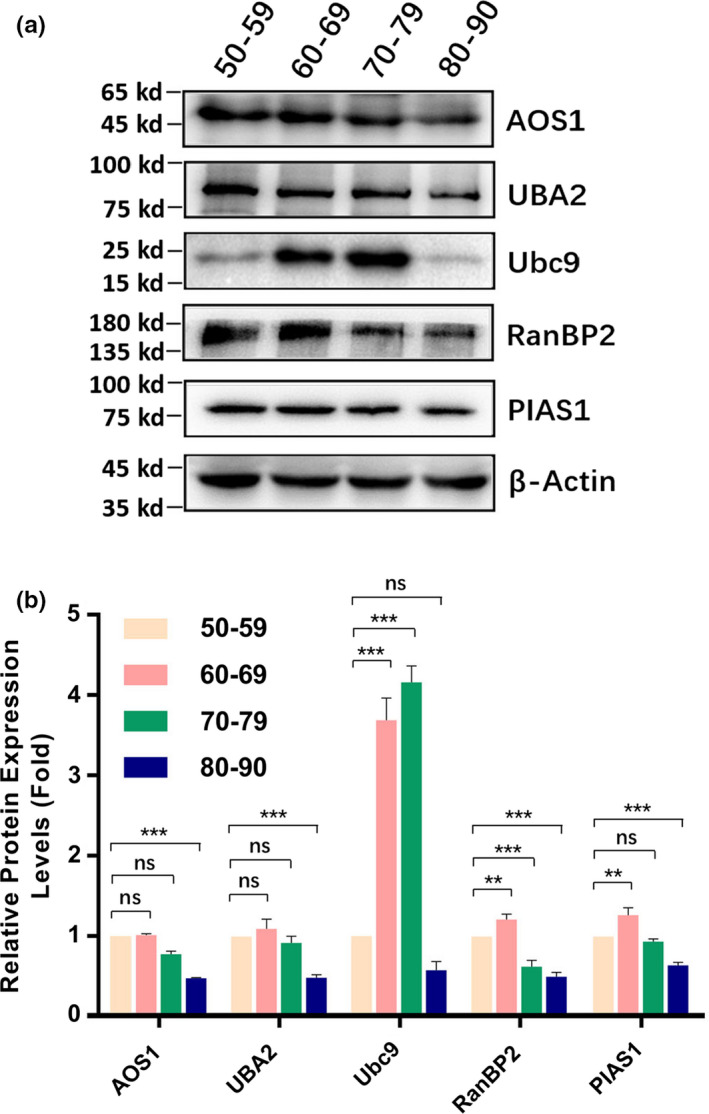
Protein levels of SUMOylation ligase 1 (AOS1 and UBA2), 2 (Ubc9), and 3 (RanBP2 and PIAS1) in human cataractous lens epithelial samples of different age groups. The capsular epithelia were isolated at surgery from individuals with different ages (31 patients of 50–59 years old, grouped as 50s, 38 patients of 60–69 years old grouped as 60s, 37 patients of 70–79 years old, grouped as 70s, and 27 patients of 80–89 years old grouped as 80s; Tables S1–S4). The capsular samples were pooled together according to their age assignments as indicated above and then used for the extraction of total proteins. Western blot analysis of three ligases was conducted as described in the Experimental Procedures. (a) Western blot analysis of three ligases in samples of different age groups as indicated in the figure. (b) Semi‐quantification of the Western blot results in Figure 1a. ns, not significant, *p < 0.05, **p < 0.01, ***p < 0.001

As shown in Figure 1a,b, among the three ligases, Ubc9, RanBP2, and PIAS1 were significantly increased from 50s to 60s. The most striking change was ligase 2 (Ubc9) with nearly threefold increase. From 60s to 70s, Ubc9 was further increased over 3.3‐fold compared with that in 50s, and RanBP2 was decreased compared with that in 50s. From 70s to 80s, ligases 1 and 3 were significantly decreased compared with those in 50s. Automated Western blot (AWB) analysis revealed that all ligases except for Ubc9 were increased compared with the normal human lenses (Figures S1–S3). Furthermore, the protein levels in cataract patients displayed clear gender difference. Most male patients displayed higher levels of ligases than female patients did (Figures S1–S3). Together, in normal human lens, all ligases except for Ubc9 displayed age‐dependent decrease. From normal human lenses to cataractous lenses, all ligases but Ubc9 showed age‐dependent increase. In cataractous lenses from 50s to 60s, ligases 2 and 3 were increased. While Ubc9 was further increased, RanBP2 was slightly decreased from 60s to 70s. All ligases were clearly decreased from 70s to 80s. The age‐dependent changes of all ligases in cataract patients of different age groups are shown in Table 1.

**TABLE 1:** Summary of age‐dependent changes in sumoylation ligases, de‐sumoylation enzymes, and their substrates in cataract samples of different age groups^a^

Sumoylation Enzymes and Substrates	50s^b^	60s	70s	80s
Sumoylation Ligases				
AOS1^c^	*	*	−	− −
UBA2^c^	*	+	−	− −
Ubc9^d^	*	+++	+++	− −
RanBP2	*	+	− −	− −
PIAS1^c^	*	++	−	− −
De‐sumoylation Enzymes				
SENP1	*	+	+	−
SENP2	*	−	*	++
SENP3^c^	*	−	− −	− − −
SENP5	*	+++	++	− −
SENP6	*	+	++	+++
SENP7^c^	*	++	++	−
SENP8^c^	*	++	+++	− − −
SUMO1‐ or SUMO2/3‐Conjugated Substrates				
SUMO1	*	++	+	*
SUMO2/3	*	++	+++	− −
p46 Pax6^c^	*	−	−	−
p32 Pax6	*	+++	− −	− − −
Sumo‐p32 Pax6	*	−	−	−

^a^ The summary data are derived from Western blot analysis of pooled cataract capsular epithelial samples.

^b^ The levels of all samples from the patients of 50s age group are used as references. * means no change compared with the level of the same sample in the 50s; +, ++, and +++ mean increase between 0.1% and 24.99%, 25% and 50%, and >50%, respectively; −, −−, and −−− mean decrease between 0.1% and 24.99%, 25% and 50%, and >50%, respectively.

^c^ Automated Western immunoblotting analysis (AWIA) of individual samples from normal human lenses and cataractous lenses reveals that Aos1, UBa2, PIAS1, SENP3. SENP7, SENP8, and p46 Pax6 are significantly increased in cataractous lenses (Figures S1, S3–S6).

^d^ AWIA of individual samples from normal human lenses and cataractous lenses reveals that Ubc9 is significantly decreased in cataractous lenses (Figure S2).

### 2.2 Protein levels of de‐sumoylation enzymes (SENPs) in cataractous lenses of different age groups

**FIGURE 2:**
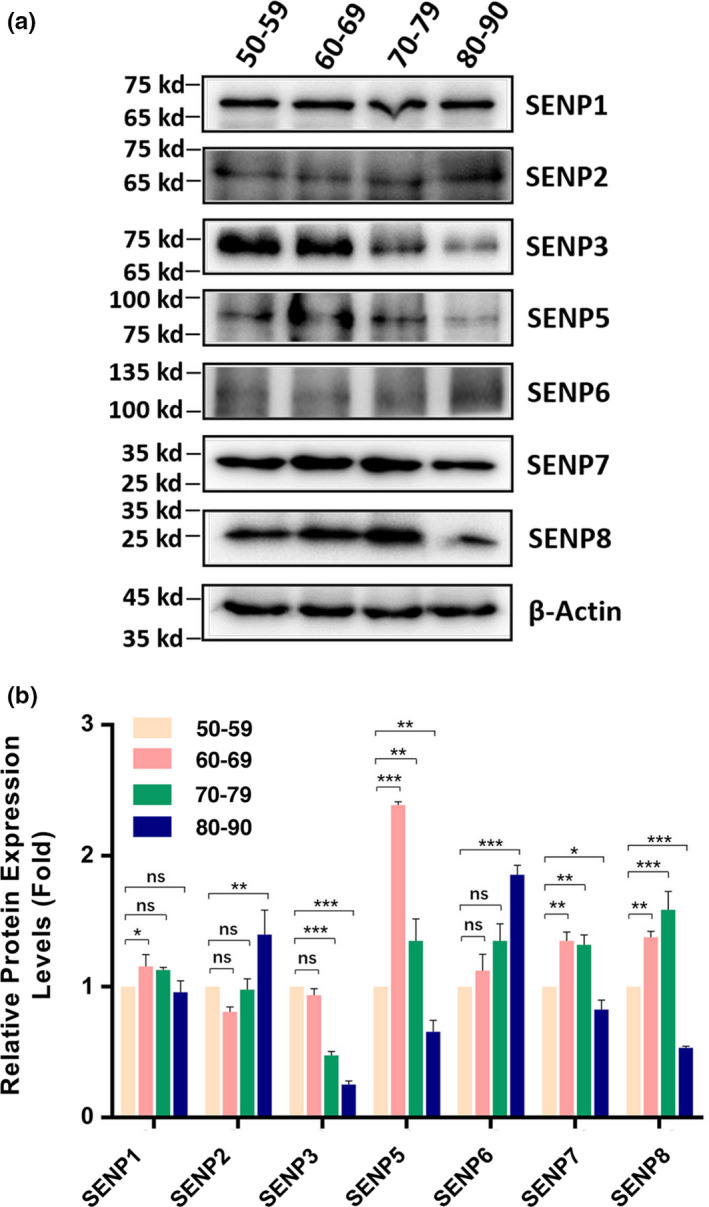
Protein levels of de‐sumoylation enzymes (SENP1, SENP2, SENP3, SENP5, SENP6, SENP7, and SENP8) in human cataractous lens epithelial samples of different age groups. The capsular epithelia were isolated at surgery from individuals with different ages as described in Figure 1. The capsular samples were pooled together according to their age assignments as indicated in Figure 1 and then used for the extraction of total proteins. Western blot analyses of seven SENPs were conducted as described in the Experimental Procedures. (a) Western blot analyses of seven SENPs in samples of different age groups as indicated in the figure. (b) Semi‐quantification of the Western blot results in Figure 2a. ns, not significant, *p < 0.05, **p < 0.01, ***p < 0.001

For SENPs, we observed many different patterns (Figure 2). SENP1 was slightly increased from 50s to 60s, stayed at similar level to that of 50s from 50s to 80s. For SENP2, it remained at similar level from 50s to 70s and then clearly increased from 70s to 80s. SENP3 displayed age‐dependent decrease from 50s and 60s to 80s. For SENP5, it was significantly increased from 50s to 60s and remained at a relatively high level from 60s to 70s but clearly decreased from 70s to 80s. SENP6 showed age‐dependent increase. SENP7 and SENP8 displayed similar patterns of changes: increased from 50s to 60s, remained unchanged or slightly increased from 60s to 70s, and clearly decreased from 70s to 80s. Together, we observed that different SENPs displayed different age‐dependent changes. From normal human lenses to cataractous lenses, AWB revealed that SENP3, SENP7, and SENP8 (other SENPs cannot be detected with 0.9 μg protein) were increased (Figures S4–S6). Also, male cataract patients had higher levels of SENP3, SENP7, and SENP8 than female patients (Figures S4–S6). The age‐dependent changes of all de‐sumoylation enzymes in cataract patients of different age groups are shown in Table 1.

### 2.3 Global SUMO1‐ and SUMO2/3‐conjugated patterns of total proteins in cataractous lenses of different age groups

**FIGURE 3:**
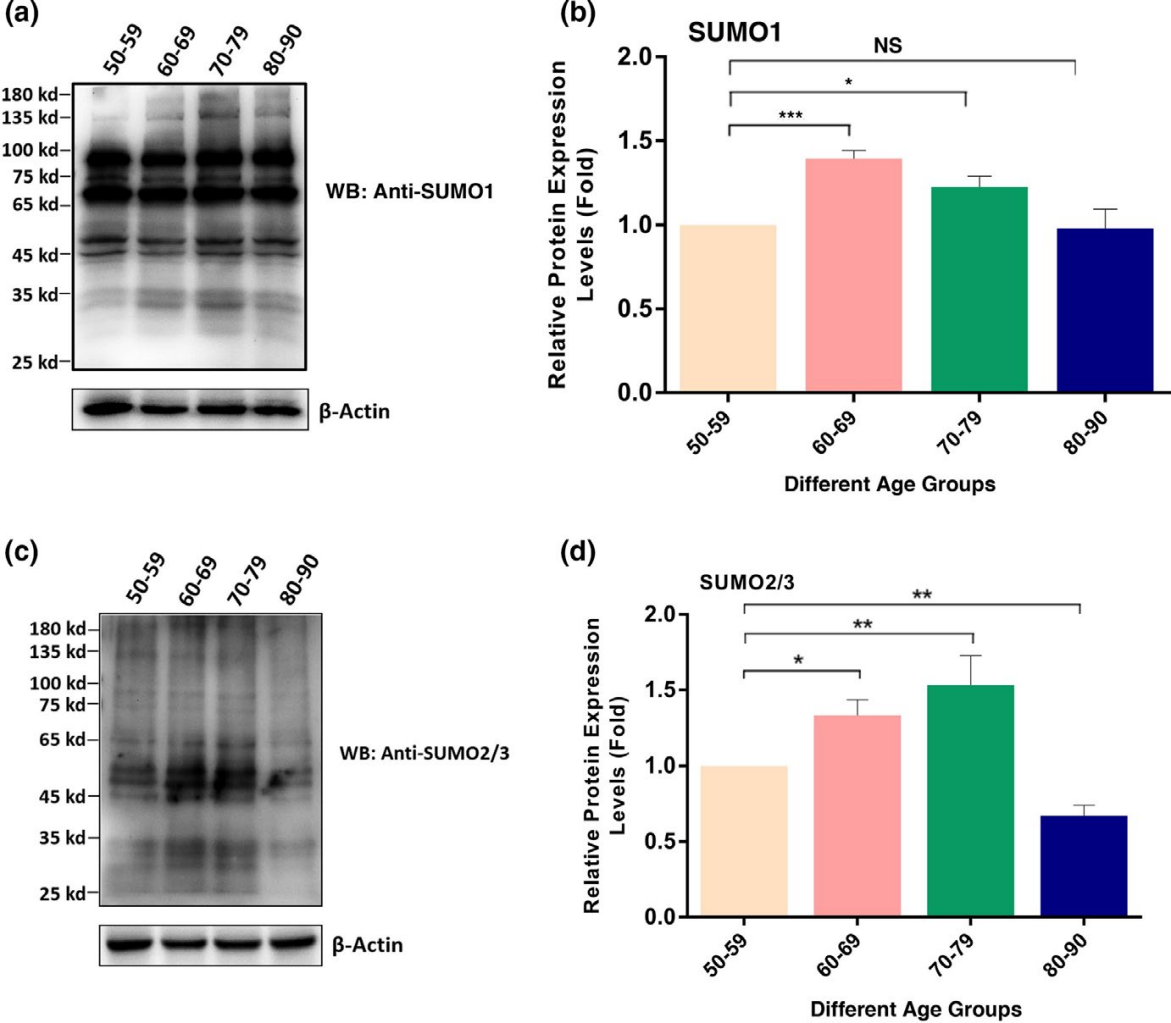
Global SUMO1‐conjugated (a and b) and SUMO2/3‐conjugated (c and d) patterns of the total proteins in human cataractous lens epithelial samples of different age groups. The capsular epithelia were isolated at surgery from individuals with different ages as described in Figure 1. The capsular samples were pooled together according to their age assignments as indicated in Figure 1 and then used for the extraction of total proteins. Western blot analysis of SUMO1‐conjugated (a and b) and SUMO2/3‐conjugated (c and d) total proteins was conducted as described in the Experimental Procedures. (a) Western blot analysis of total proteins with SUMO1 conjugation in samples of different age groups as indicated in the figure. (b) Semi‐quantification of the Western blot results in (a). (c) Western blot analysis of total proteins with SUMO2/3 conjugation in samples of different age groups as indicated in the figure. (d) Semi‐quantification of the Western blot results in Figure 4a. NS, not significant, *p < 0.05, **p < 0.01, ***p < 0.001

After analysis of the age‐dependent changes in sumoylation enzyme system, we next examined the global sumoylation patterns of total proteins in capsular epithelia of human cataractous lenses of different age groups. First, we determined the SUMO1 conjugation patterns of total proteins in these samples. As shown in Figure 3a,b, two major changes were observed from 50s to 80s. First, the SUMO1‐conjugated proteins in both higher molecular weights (>100 kD) and lower molecular weights (<35 kD) were clearly increased from 50s to 70s and became most visible in 70s. They resumed to the level of 50s from 70s to 80s. Second, for the SUMO1‐conjugated proteins between 35 and 100 kD, they were relatively stable. Thus, SUMO1 conjugation of the lens proteins in the capsular epithelia displayed first increased and then resumed to the level of 50s. Similar to SUMO1, global SUMO2/3 conjugations of total lens proteins in the capsular epithelia of different age groups were steadily increased from 50s to 70s and then decreased from 70s to 80s (Figure 3c,d. The age‐dependent changes of both SUMO1‐ and SUMO2/3‐conjugated substrates in cataract patients of different age groups are shown in Table 1.

### 2.4 Protein level and sumoylation status of Pax6 in normal and senile cataractous lenses of different age groups 

**FIGURE 4:**
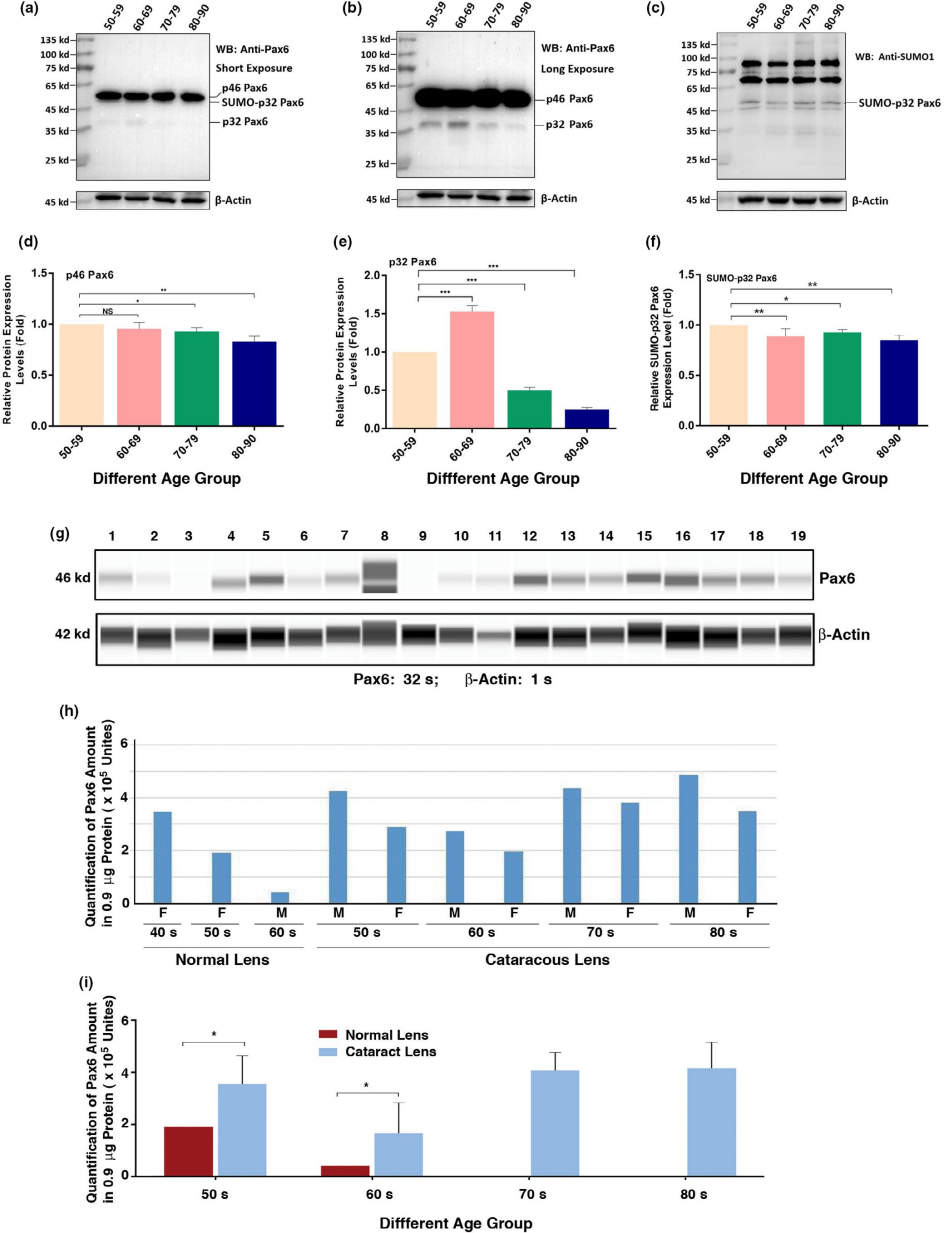
Protein levels of p46 and p32 Pax6 in human cataractous lens epithelial samples of different age groups. The capsular epithelia were isolated at surgery from individuals with different ages as described in Figure 1. The capsular samples were pooled together according to their age assignments as indicated in Figure 1 and then used for the extraction of total proteins. Western blot analysis of p46 Pax6 was conducted as described in the Experimental Procedures. (a) Western blot analysis of p46 Pax6 in samples of different age groups as indicated in the figure. (d) Semi‐quantification of the p46 Pax6 band in the Western blot of (a). (b) Western blot analysis of p32 Pax6 in samples of different age groups as indicated in the figure. The same blot in (a) was exposed 2x more time to reveal the 32 kd p32 Pax6 band. (e) Semi‐quantification of the p32 Pax6 band in the Western blot of (b). (c) Western blot analysis of SUMO1‐conjugated p32 Pax6 in samples of different age groups as indicated in the figure. (f) Semi‐quantification of the SUMO1‐conjugated p32 Pax6 band in the Western blot of (c). (g–i), The automated Western immunoblot (AWI) analysis of Pax6 in normal and cataractous lenses of different age groups. AWI was performed on a PeggySue (ProteinSimple) as described recently (Dahl et al., 2016). Briefly, each sample was loaded with 0.9 μg total protein and then analyzed with the Size Separation Master Kit and Split Buffer (12–230 kDa) according to the manufacturer's standard instruction using anti‐Pax6 antibody (for antibody information, see Experimental Procedures) with a dilution factor of 1:100. The Compass software (Protein Simple, version 4.1.5) was used to program the PeggySue‐robot and for presentation (g) and quantification (h and i). Output Western blot style data (g) were displayed with exposure time indicated, and the quantification data (h and i) were displayed from the software‐calculated average of seven exposures (1–512 s). (h) Quantification results show gender difference. Each bar represents an average of two samples for cataract lenses but one sample for normal human lens. (i) Quantification results show age difference. NS, not significant, *p < 0.05, **p < 0.01, ***p < 0.001

After analyzing the sumoylation patterns of total proteins in the capsular epithelia of different age groups of cataract patients, we next sought to analyze an individual protein. Since we have previously demonstrated that SUMO1 conjugation of Pax6 is necessary to activate its functions during lens development (Yan et al., 2010), we next sought to determine whether the Pax6 level and sumoylation status display dynamic changes in cataract patients of different age groups. As shown in Figure 4a, d, the p46 Pax6 level remained relatively strong in all age groups and displayed no significant changes from 50s to 60s, and then decreased between 5% and 10% from 60s to 70s, and from 70s to 80s. The p46 Pax6 in senile cataractous samples of different age groups appears un‐conjugated by SUMO1 or SUMO2/3 (Figure 4a, b).

A much longer exposure of Figure 4a allowed us to observe the dynamic changes of the p32 Pax6. As shown in Figure 4b, e from 50s to 60s, p32 Pax6 was increased about 0.5‐fold and then steadily decreased from 60s to 80s over 50% (the protein samples in these studies moved a little slower due to phosphorylation of the serine/threonine residues in the PST domain of the C‐terminal (Yan et al., 2007) as reflected by the fact that p32 Pax6 is above 35 kD marker). From Figure 4a, we observed a weak band of 43 kD below the p46 Pax6, and this is the SUMO1‐conjugated p32 Pax6 as confirmed with anti‐SUMO1 antibody from the result of Figure 4c. Both anti‐Pax6 and anti‐SUMO1 can detect the same 43 kD band (Figure 4a, c). A semi‐quantitative analysis of the sumoylated p32 Pax6 (Figure 4f) revealed that the SUMO1‐conjugated p32 Pax6 is much stable than the non‐conjugated p32 Pax6 (Figure 4b, e). From 50s to 80s, the SUMO1‐conjugated p32 Pax6 was decreased in less than 20%, compared to more than 70% in non‐conjugated p32 Pax6 (Figure 4c,f). Thus, SUMO1 conjugation increases the stability of p32 Pax6. AWB analysis revealed that compared with age‐matched normal lenses, the Pax6 level was much increased in cataract patients (Figure 4g,i). Moreover, male cataract patients displayed a higher level of Pax6 than female patients did (Figure 4h). The age‐dependent changes of different isoforms of Pax6 in cataract patients of different age groups are shown in Table 1.

### 2.5 Protein levels and sumoylation status of p46 and p32 Pax6 in cataract patients with other complications

**FIGURE 5:**
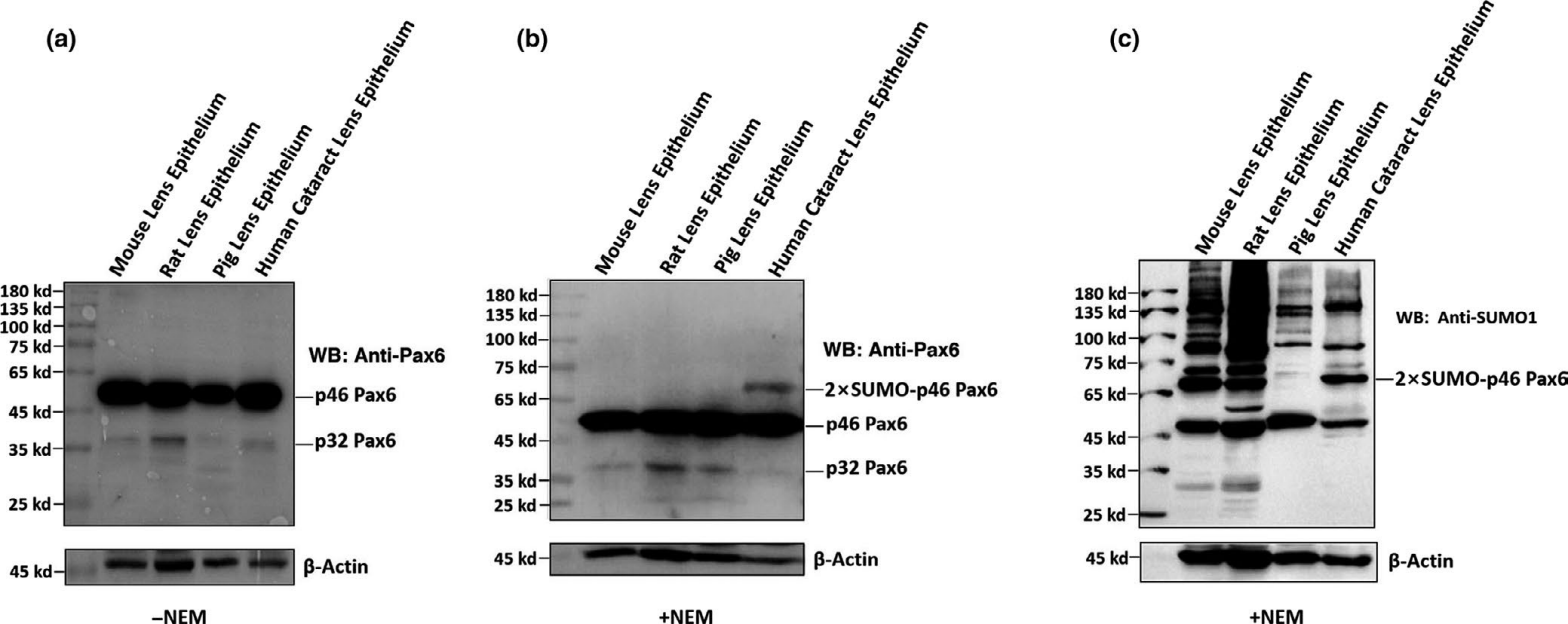
Analysis of expression and sumoylation patterns of p46 Pax6 and p32 Pax6 in complicated cataractous lens epithelial samples of different age groups with cataract and also other syndromes. The capsular epithelia were isolated during cataractous surgical operations from individuals with different ages and complications (Table S5). The capsular samples were pooled together and then used for the extraction of total proteins in the absence of inhibitors for de‐sumoylation (−NEM, Figure 5a) or the presence of inhibitors for de‐sumoylation (+NEM, Figure 5b,c). Western blot analysis was conducted as described in the Experimental Procedures. (a) Western blot analysis of p46 and p32 Pax6 in samples of different age groups with other complications pooled together as indicated in Table S5. The capsular epithelia used as control were isolated from transparent lenses of mice, rats, and pigs. The protein extraction was conducted in the absence of the SENP inhibitor (−NEM). (b) Western blot analysis of and p46 and p32 Pax6 in samples of different age groups with other complications pooled together as indicated in Table S5. The capsular epithelia used as control were isolated from transparent lenses of mice, rats, and pigs. The protein extraction was conducted in the presence of the SENP inhibitor (+NEM). (c) The same blot in Figure 5b was stripped off the anti‐Pax6 antibody with stripping buffer and then hybridized with anti‐SUMO1 antibody. After 3X washes with TPST, the blot was incubated with the secondary antibody for 45 min. After 2X washes with TPST, and 1X with TBS, and then exposed to reveal the presence of the 68 kD di‐sumoylated p46 Pax6 band, and the 43 kd sumoylated p32 Pax6 band

After comparative analysis of the p46 and p32 Pax6 in senile cataractous lenses of different age groups, we next examined the relative expression levels and sumoylation patterns of both p46 and p32 Pax6 in cataract patients with other complications (Table S5). As shown in Figure 5, in the pooled epithelial cell samples from complicated cataract patients of 50s to 90s, p46 Pax6 was dominant (the rightmost lane of Figure 5b), and p32 Pax6 was expressed at a very weak level. Most strikingly, we observed a di‐sumoylated p46 Pax6 band of 68 kD. This band was also easily detected by anti‐SUMO1 antibody (Figure 5c). In addition, Figure 5c also revealed the presence of the 43 kD SUMO1‐conjugated p32 Pax6. When we conducted this study, we could not get transparent human lenses of the same age groups as control, capsular epithelial samples from adult mouse, rat, and pig lenses were used (the left three lanes of the blot in Figure 5). Clearly, we did not observe the same di‐sumoylated p46 Pax6 band in these control lenses (Figure 5b,c) or in senile cataractous lenses (Figure 4). Thus, our results revealed the presence of the di‐sumoylated p46 Pax6 only in complicated cataract patients.

### 2.6 Glucose oxidase (GO) treatment induces upregulation of Pax6 expression and apoptosis of capsular lens epithelial cells followed by cataractogenesis

**FIGURE 6:**
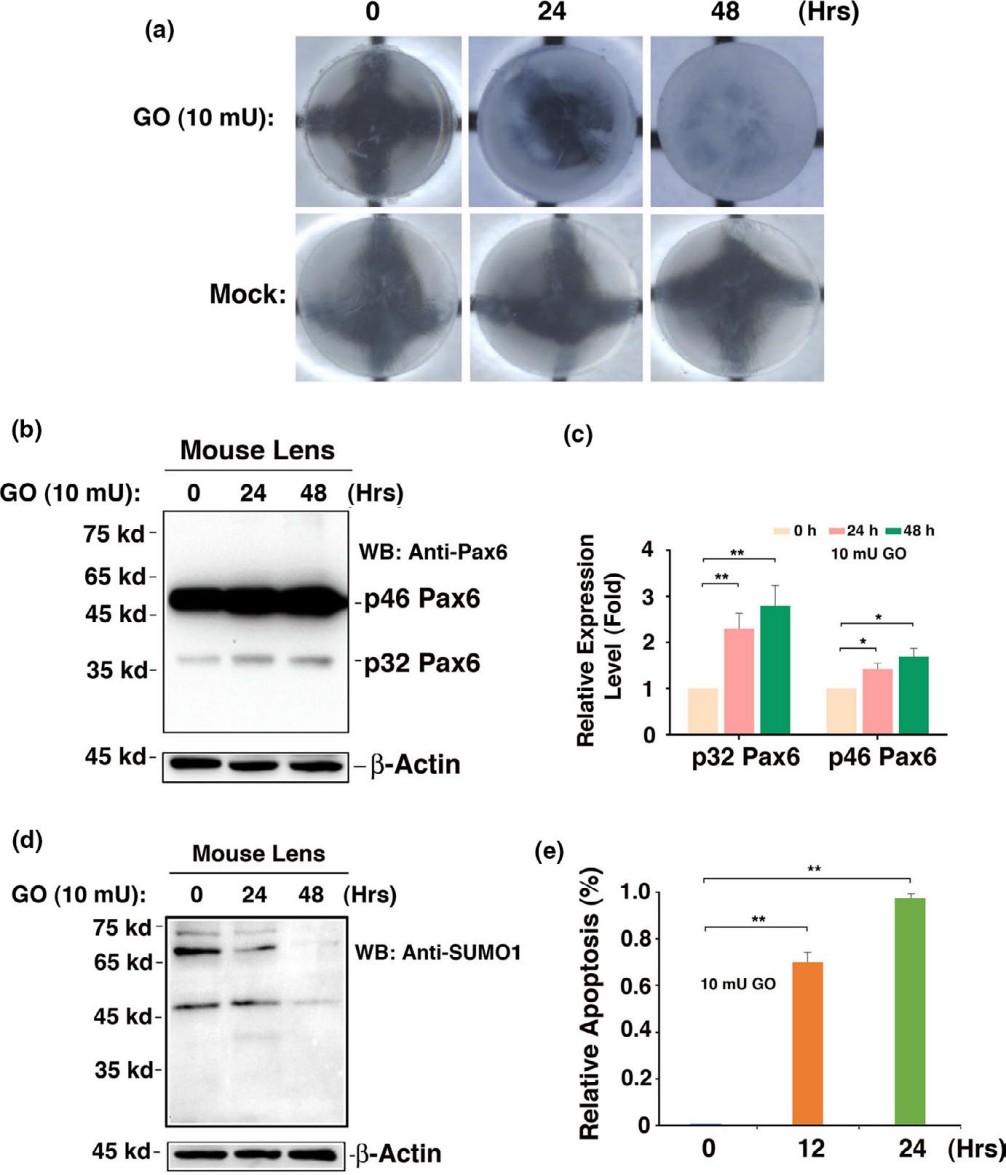
Glucose oxidase (GO) treatment‐induced in vitro cataractogenesis is preceded by the upregulation of Pax6 and the induction of apoptosis. (a) Treatment of mouse lenses by 10 mU/ml GO caused in vitro cataractogenesis. Note that the transparent lens became opaque in most area after 24‐hr treatment and complete opaque after 48‐hr treatment by 10 mU/ml GO. (b) Western blot analysis of Pax6 in mouse lens epithelia after treatment with 10 mU glucose oxidase for 0–48 hr as indicated. (c) Semi‐quantification of the Western blot results in (d). (d) Western blot analysis of SUMO1‐conjugated proteins in mouse lens epithelia after treatment with 10 mU glucose oxidase for 0–48 hr as indicated. (e) Apoptotic rate of epithelial cells in mouse lenses after 10 mU/ml GO treatment for 0–24 hr. Note that a 12‐hr treatment induced nearly 80% apoptosis and increased to 100% apoptosis after 24‐hr treatment. Thus, GO‐induced apoptosis preceded the in vitro cataractogenesis (a). **p < 0.01

Our previous studies have shown that oxidative stress‐induced apoptosis acts as a major cellular mechanism for cataractogenesis (Li et al., 1995; Li & Spector, 1996). The presence of strong p46 Pax6 expression in capsular epithelia of cataract patients of different age groups prompts us to explore its possible functions in the adult lenses. For this purpose, we dissected 80 lenses from 4‐week‐old mice. After 12‐hr incubation in medium 199, 63 transparent lenses were selected. Among which 30 were used as control and 33 were treated with 10 mU/ml GO to generate oxidative stress (100 μM hydrogen peroxide in a 24‐hr period), the morphological, cellular, and molecular changes were analyzed at 0, 24, and 48 hr post‐GO treatment. As shown in Figure 6a, a treatment with 10 mU/ml GO for 24–48 hr induced in vitro cataract development. Within 48 hr, the lens became completely opaque, while the control lens remained completely transparent during the same period of culture. During the development of in vitro cataract, apoptosis of the capsular epithelial cells was substantially activated, leading to nearly 80% and 100% apoptosis after 12‐ and 24‐hr treatment (Figure 6e), which is consistent with our previous study (Li et al., 1995). More importantly, we observed that GO treatment also upregulated expression of both p46 and p32 Pax6 (Figure 6b,c). GO treatment did not induce Pax6 sumoylation (Figure 6d), implying that non‐sumoylated Pax6 may be implicated in regulation of apoptosis. The GO‐induced upregulation of Pax6 contributes to activated apoptosis and cataractogenesis (Fu, Liu, Wang & Li, 2019).

## 3 DISCUSSION

In the present study, we have demonstrated the following: (a) Sumoylation ligases are upregulated in cataract patients from 50s to 60s, remains at a high level in those from 60s to 70s, but all downregulated in those from 70s to 80s in comparison with that in 50s; (b) de‐sumoylation enzymes, SENPs, displayed differential changes among all enzymes in cataract patients from 50s to 80s; and (c) SUMO1‐ and SUMO2/3‐conjugated proteins are upregulated in cataract patients from 50s to 70s. For cataract patients from 70s to 80s, both SUMO1‐ and SUMO2/3‐conjugated proteins are decreased with differential levels; (d) among the non‐sumoylated p46 and p32 Pax6 isoforms in cataract patients of different age groups, the stability of p46 Pax6 is much stronger than that of p32 Pax6; (e) in senile cataract patients, p32 Pax6 displays clear sumoylation, and the sumoylated p32 Pax6 (43 kD) displays much better stability than the non‐sumoylated p32 pax6; (f) in the complicated cataract patients, p46 Pax6 is clearly sumoylated with dual SUMO conjugations and thus appears as a 68 kd sumoylated isoform; in these patients, the sumoylated 43 kD p32 Pax6 also exists; (g) AWB analysis reveals that all sumoylation enzymes analyzed except for Ubc9 were increased in cataractous lenses compared with those in age‐matched normal lenses; moreover, male patients had higher levels of sumoylation enzymes and Pax6 than female patients did; (h) oxidative stress‐induced apoptosis followed by cataractogenesis is linked with upregulated expression of Pax6 in mouse lenses. From these studies, we conclude that Ubc9, SENP6, and p32 Pax6 can be used as molecular markers for simple senile cataracts. In contrast, di‐sumoylated p46 Pax6 can be used as a molecular marker for complicated cataracts. Together, our results have identified the molecular signatures for both senile and complicated cataracts and demonstrate the presence of linkage between sumoylation and cataractogenesis.

### 3.1 Differential protein levels of various ligases and de‐sumoylation enzymes in the cataractous lenses of different age groups are linked with aging control

Our results revealed that during aging of the cataractous patients from 50s to 70s, the majority of the ligases are clearly upregulated (Figure 1). Such upregulation causes the enhanced sumoylation patterns of the total proteins in the capsular epithelial cells conjugated by either SUMO1 or SUMO2/3 (Figure 3). Although the majority of the targets have not been elucidated in the ocular lenses, previous studies from others in non‐lens systems have shown that sumoylation is implicated in aging control through modulation of different targets.

First, sumoylation is important in control of telomere stability and DNA repair process to resist aging. It is well established that mutations in the dyskerin gene (DKC1) cause X‐linked dyskeratosis congenita (DC), a rare and fetal premature aging syndrome characterized by defective telomere maintenance (Devriendt et al., 1997). Dyskerin is a highly conserved nucleolar protein and a component of the human telomerase complex that is essential for human telomerase RNA (hTR) stability (Shay & Wright, 2019). Brault, Lauzon, and Autexier (2013) have shown that dyskerin can be sumoylated at K39 and K43, and both K39R and K43R mutants display impaired hTR accumulation, telomerase activity, and telomere maintenance. Our previous studies have shown that telomere shortening is linked with cataractogenesis (Huang et al., 2005).

In addition, sumoylation is also necessary for maintaining a normal DNA repair system. Cockayne syndrome (CS) is a severe neurodegenerative and premature aging autosomal‐recessive disease, caused by inherited mutations in the CSA and CSB genes, leading to defects in transcription‐coupled nucleotide excision repair and consequently hypersensitivity to ultralight (UV) irradiation (Groisman et al., 2006). Liebelt et al. (2020) have demonstrated that during UV irradiation, CSB is the most dynamically sumoylated substrate of the repair complex, which is necessary for its recruitment into the UV‐induced damaging site and subsequently helps the maintenance of RNA synthesis as well as efficient transcription‐coupled nucleotide excision repair (Liebelt et al., 2020). In the ocular lens, DNA damage is detected in the capsular epithelial cells of senile cataracts or stress‐induced cataract lenses (Friedrich, Wang, Schey, & Truscott, 2019; Garland, 1990; Kleiman & Spector, 1993; Michael, Vrensen, van Marle, Löfgren, & Söderberg, 2000; Uwineza, Kalligeraki, Hamada, Jarrin, & Quinlan, 2019). Together, these studies show that sumoylation plays a role against aging. Lack of normal sumoylation causes deficient telomere maintenance and DNA repair system, leading to genome instability and lens pathology.

On the other hand, sumoylation is implicated in promotion of aging. The most striking examples are the studies on the neurological diseases (Bao et al., 2018; Luo et al., 2014; Rott et al., 2017; Steffan et al., 2004). These studies have shown that sumoylation plays a causing role in several age‐related neurological diseases. For example, amyloid beta (Aβ) is a major pathological marker in Alzheimer's disease (AD), which is principally regulated by the rate‐limiting β‐secretase (BACE1) cleavage of amyloid precursor protein (Bao et al., 2018). Bao et al. demonstrated that BACE1 is predominantly SUMOylated at K501 residue, which escalates its protease activity and stability and subsequently increases Aβ production, leading to cognitive defect seen in the AD mouse model. Overexpression of wild‐type BACE1, but not the K501R mutant, facilitates senile plaque formation and aggravates the cognitive deficit seen in the APP/PS1 AD mouse model (Bao et al., 2018).

In Parkinson's disease (PD), α‐synuclein accumulation is a pathological hallmark (Rott et al., 2017). Rott et al. identified that SUMOylation acts as a major mechanism that counteracts ubiquitination by different E3 ubiquitin ligases and regulates α‐synuclein degradation. They further showed that PIAS2 promotes SUMOylation of α‐synuclein, leading to a decrease in α‐synuclein ubiquitination by SIAH and Nedd4 ubiquitin ligases, and causing its accumulation and aggregation into inclusions. Besides AD and PD, sumoylation is also involved in the pathogenesis of Huntington's disease (HD).

Sumoylation is also implicated in modulation of major signaling pathways to promoting aging. Li et al. (2006) have previously shown that sumoylation can modulate major aging control pathways mediated by two important tumor suppressors, p53 and RB. Yu et al. (2018) have recently demonstrated that sumoylation of the major signaling kinase, CDK9, can suppress global transcription to promote aging.

In the present study, we have shown that sumoylation of total proteins in the capsular epithelial cells of cataractous lenses is significantly enhanced from 50s to 70s (Figure 3). Whether sumoylation modulates the above signaling pathways and targets to promote lens aging and pathology is currently under investigation. Nevertheless, our demonstration that sumoylation enhances the stability of p32 Pax6 and Pax6 is increased from normal lenses to cataractous lenses indeed support the conclusion that sumoylation could promote lens pathology (see discussion below). We also observed obvious gender difference in the levels of sumoylation ligases, de‐sumoylation enzymes, and Pax6 (Figure 4g–i, Figures S1–S6). Considering that Pax6 increase is linked with aging and cataractogenesis, we speculate that the above gender difference suggests that female individuals may age more slowly than male individuals do. At present, we do not know what mechanisms cause the observed gender difference.

### 3.2 Sumoylation of p32 Pax6 enhances its stability in cataractous lenses of different age groups

Pax6 is a highly conserved transcription factor, which acts as a master regulator controlling eye and brain development in humans, mice, zebrafish, and Drosophila (Callaerts, Halder, & Gehring, 1997; Glaser, Walton, & Maas, 1992; Hill et al., 1991). The conserved essential amino acid sequence of Pax‐6 proteins in different species predicts its fundamental roles in controlling development of these organisms (Czerny, Schaffner, & Busslinger, 1993; Epstein, Cai, Glaser, Jepeal, & Maas, 1994; Wilson, Sheng, Lecuit, Dostatni, & Desplan, 1993). Indeed, expression of exogenous Pax6 in *Drosophila* induces generation of ectopic compound eye in different tissues (Halder, Callaerts, & Gehring, 1995). Furthermore, haploid insufficiency or deletion of the Pax‐6 gene leads to various ocular diseases including aniridia, cataracts, and glaucoma (Glaser, Jepeal, Edwards, Young, & Favor, 1994). A homozygous mutation in Pax‐6 causes lethality at birth, with severe brain defects and the absence of eyes and nose in humans and mice (Glaser et al., 1992; Hill et al., 1991).

At the molecular level, Pax‐6 functions primarily to mediate the commitment of the ectoderm above the optic vesicle into the lens ectoderm and also to promote formation of the lens vesicle (Ashery‐Padan, Marquardt, Zhou, & Gruss, 2000; Li, Yang, Jacobson, Pasko, & Sundin, 1994). Pax‐6 controls transcriptional expression of genes encoding both transcription factors such as c‐Maf, Sox‐1, and Prox‐1, and also lens structural proteins including α‐, β‐, and γ‐crystallins (Ashery‐Padan et al., 2000; Chauhan, Reed, Zhang, Duncan, & Kilimann, 2002; Cvekl, Sax, Li, McDermott, & Piatigorsky, 1995; Duncan, Kozmik, Cveklova, Piatigorsky, & Cvekl, 2000; Li et al., 1994; Shaham, Smith, Robinson, Lang, & Ashery‐Padan, 2009). Previous studies have demonstrated the presence of at least four forms of Pax‐6 in quail cellular extracts with molecular masses of 48, 46, 43, and 32 kD, which were named “p48,” “p46,” “p43,” and “p32,” respectively (Carriere, Plaza, Martin, Quatannens & Bailly, 1993). These isoforms vary from each other in their N‐terminal structures. The common p46 isoform has a 128‐amino‐acid PD at the N‐terminus and a 56‐amino‐acid HD in the central region which bind specifically to different DNA sequences, P6CON, and P3(TAAT/ATTA), respectively (Chauhan et al., 2002; Cvekl et al., 1995; Czerny et al., 1993; Duncan et al., 2000; Epstein et al., 1994; Richardson, Cvekl, & Wistow, 1995; Shaham et al., 2009; Wilson et al., 1993). At the C‐terminus, a conserved domain rich in proline (P), serine (S), and threonine (T) residues exists in all Pax‐6 isoforms and thus is named the “PST” domain. This domain mediates activation of Pax‐6 through phosphorylation by p38 MAP kinase and homeodomain‐interacting protein kinase 2 (Kim, Noh, et al., 2006; Mikkola, Bruun, Bjorkoy, Holm, & Johansen, 1999). Several phosphorylation sites have been identified in the PST domain of human and zebrafish Pax‐6. Our previous studies have shown that both p32 and p46 Pax‐6 isoforms are subjected to negative regulation by protein serine/threonine phosphatase‐1 (PP‐1) in vitro and in vivo (Yan, Liu, Qin, Liu, & Chen, 2007). Furthermore, our previous studies have shown that activation of the p32 Pax6 requires SUMO1‐mediated sumoylation at the K91 residue, and the activated p32 Pax6 can control the downstream genes to regulate brain and eye development in the early embryonic stages (Yan et al., 2010). Our previous study, however, left an important question unanswered: Whether sumoylation changes the stability of p32 Pax6? In the present studies through analysis of the changes of the non‐sumoylated and sumoylated p32 Pax6 in different groups of cataract patients, we clearly demonstrated that the non‐sumoylated p32 Pax6 is increased in those from 50s to 60s. However, from 60s to 80s, p32 Pax6 is sharply downregulated more than 80% (Figure 4b,e). On the other hand, SUMO1‐conjugated p32 Pax6 displayed less than 15% downregulation from 50s to 80s (Figure 4c,f). Thus, our results reveal that SUMO1‐conjugated sumoylation enhances the stability of p32 Pax6. Different from the p32 Pax6, the p46 Pax6 shown much better stability. From 50s to 80s, less than 20% p46 Pax6 is degraded (Figure 4a,d). Moreover, we could not clearly detect the sumoylation of p46 Pax6 in the epithelial cell samples of the simple senile cataractous lenses, which is different from the condition of the complicated cataractous lenses where we observed its di‐sumoylation (see discussion below). How could the enhanced sumoylation of p32 Pax6 promote lens pathology? Our results further show that during stress‐induced cataractogenesis, expression of Pax6 was upregulated (Figure 6). The upregulated Pax6 can promote apoptosis through control of the apoptosis‐related genes. One of such gene is Birc7, a member of IAP family (Fu et al., 2019). Thus, our results demonstrate that sumoylation control of Pax6 activity not only regulates lens development but also contributes to lens pathology.

### 3.3 Molecular signature for senile and complicated cataract patients.

The ocular lens is an excellent organ to study aging because its simplicity in structure with only two types of cells, the anterior single layer of epithelial cells and the differentiating or differentiated fiber cells (Yan, Liu & Li, 2006). In addition, it does not have any distribution of vascular and nerve systems. Furthermore, we have previously shown that the lens epithelial cells have the similarly conserved signaling pathways but distinct functions (Li, Liu, Mao, Xiang & Wang, 2005). For example, the stress‐activated MAPK kinases assembling into Ras/Raf/MEK/ERK pathway confer survival in non‐lens cells (Xia, Dickens, Raingeaud, Davis & Greenberg, 1995) but mediate stress‐induced apoptosis in lens epithelial cells (Li et al., 2005).

During aging of the ocular lens, various types of molecular changes contribute to cataractogenesis. For example, extensive changes have been reported in major lens structure proteins, members of different families of crystallins including α‐, β‐, and γ‐crystallins (Friedrich, Wang, Oakley, Schey, & Truscott, 2017; Quinlan & Hogg, 2018; Su et al., 2012; Truscott & Friedrich, 2016; Wang, Friedrich, Truscott & Schey, 2019; Wang, Lyons, Truscott & Schey, 2014). In this regard, Su et al. (2012) observed that autolytic cleavage of crystallins adjacent to serine residues yields about 25% of all peptides derived from αA‐, αB‐, β‐, and γS‐crystallins, and these authors suggest that the autolytic cleavage adjacent to serine residue generates the molecular signatures of long‐lived proteins in the ocular lens (Su et al., 2012). In a subsequent study, Wang et al. (2014) suggest that non‐enzymatic post‐translational modifications (PTM) of the lens structure proteins seem to be a fundamental molecular process of aging since the combination of various modifications and their accumulation with age not only affects function, but leads to crosslinking and protein aggregation, thus causing light scattering.

Besides lens crystallins, molecular changes in various enzymes responsible for cellular redox setting, cytoskeletons, and intermediate filaments induced by oxidative and other stress conditions are also linked to aging and lens pathology (Barnes & Quinlan, 2017; Fan, Monnier, & Whitson, 2017; Giblin et al., 1995; Raghavan et al., 2016; Rakete & Nagaraj, 2016; Reddy et al., 2001; Wang et al., 2017). A recent study by Wang et al. (2017) identified 74 and 50 disulfide‐forming proteins in human and mouse cataractous lenses among which a majority of proteins are redoxing enzymes. In addition, extensive oxidation was found in lens‐specific intermediate filament proteins (Wang et al., 2017).

The above molecular changes are generally caused by non‐enzymatic reactions. Different from the changes in the above‐mentioned molecules, in the present study, we reported here the aging‐related changes induced by enzymatic reactions. By analyzing the protein levels of the sumoylation ligases and de‐sumoylation enzymes, and the sumoylation patterns of total proteins in capsular epithelia from cataractous lenses of different age groups, we demonstrated that the sumoylation enzyme system displays distinct changes, and the ligases UBA2, Ubc9, and PIAS1, as well as the de‐sumoylation enzyme SENP2/6 are found upregulated from 50s to 70s. All sumoylation enzymes, however, are downregulated from 70s to 80s. Thus, these enzymes, especially Ubc9 and SENP6, can be used as molecular markers for senile cataract. Among sumoylation substrates, we have examined the protein levels of both p46 and p32 Pax6. While p46 Pax6 remains relatively stable from 50s to 80s, the p32 Pax6 is significantly downregulated from 60s to 80s. The contrast stability of p46 and p32 Pax6 can be used as another molecular marker for senile cataract.

Analysis of the pooled epithelial cell samples from complicated cataract patients of 50s to 90s revealed the presence of di‐sumoylated p46 Pax6. Such sumoylation pattern was only detected in the cataract with other complications but not in the simple senile cataract or normal animal lenses. Therefore, the di‐sumoylated p46 Pax6 can be used as the molecular marker for the complicated cataract. At present, we do not know what condition activates di‐sumoylation of Pax6 because of limitation to obtain enough complicated cataract samples of each type.

In summary, the results presented in this study reveal important changes in the protein levels of sumoylation enzyme system and sumoylation patterns of their substrates in cataract patients of different age groups. These results allow us to draw two conclusions: 1) Sumoylation is implicated in control of aging and cataractogenesis; and 2) Ubc9, SENP6, and Pax6 can be used as the molecular signature for senile cataracts, but the di‐sumoylated p46 Pax6 can be used as molecular signature for complicated cataracts.

## 4 EXPERIMENTAL PROCEDURES

### 4.1 Animals

The study was performed using 4‐week‐old C57BL/6J mice, 4‐week‐old Sprague‐Dawley (SD) rats, and 3‐month‐old pigs. Detailed information is described in the supporting Experimental Procedures.

### 4.2 Collection of lens capsular epithelia

Collection of human capsular epithelia from cataract lenses of different age groups was approved by the Institutional Review Board of the Zhongshan Ophthalmic Center (ZOC). Informed written consent was obtained from each of the cataract patients. Detailed information is described in the supporting Experimental Procedures.

### 4.3 Lens organ culture

Dissection, selection, and culture of lens organs from mice, rats, and pigs were conducted as we described before (Li et al., 1995; Li & Spector, 1996). Detailed information is described in the supporting Experimental Procedures.

### 4.4 Glucose oxidase (GO) treatment

Glucose oxidase treatment was conducted as we described (Gong et al., 2018; Li et al., 1995). Detailed information is described in the supporting Experimental Procedures.

### 4.5 Apoptosis assays

The percentage of apoptosis in GO‐treated mouse lenses were determined by CellTiter‐Glo^®^ Luminescent Cell Viability Assay Kit (G7573, Promega) as described before (Crouch, Kozlowski, Slater & Fletcher, 1993).

### 4.6 Total protein extraction and Western blot analysis

Total proteins were extracted from capsular epithelial and cultured animal lenses with using RIPA buffer, processed for Western blot analysis as we described before (Gong et al., 2014, 2018; Li et al., 1995, 2005; Li & Spector, 1996; Yan et al., 2007, 2010). Detailed information is described in the supporting Experimental Procedures.

### 4.7 Automated Western immunoblotting

The simple Western immunoblots were performed on a PeggySue (ProteinSimple) as previously described (Dahl et al., 2016). Detailed information is described in the supporting Experimental Procedures.

### 4.8 Protein solubility analysis

The protein solubility from human capsular epithelium samples of different age group was determined as described in supporting Experimental Procedures.

### 4.9 Statistical analysis

Results presented in the figures are representative of three or more independent repetitions. All data were analyzed with SPSS 17.0 software (SPSS Inc., Chicago, IL, USA). One‐way analysis of variance (ANOVA) followed by Tukey's test for multiple comparisons was used for statistical analysis. The *p*‐value <0.05 was considered statistically significant. *, **, and *** represent *p* < 0.05, 0.01, and 0.001, respectively.

## CONFLICT OF INTEREST

The authors declare no competing interests.

## AUTHOR CONTRIBUTIONS

FYL, JLF, and DW‐CL designed research. FYL, JLF, LW, QN, and ZWL performed the experiments. MH, YY, XDG, YW, YX, JWX, XBH, and LZ coordinated collection of capsular epithelial samples and involved in collection of animal lenses. MXW, WRC, BC, LXL, XYZ, XLL, DYZ, and SSH provided capsular epithelia at surgery. FYL, YZL, and DW‐CL analyzed the data and wrote the paper.

## ETHICAL APPROVAL

All authors approve this submission.

## PATIENT CONSENT STATEMENT

Patient consent has been obtained for all patients. Permission to reproduce material from other sources: Not applicable. Clinical Trial Registration: Not applicable.

## Supporting information

Supplementary Material

## Data Availability

All data are available upon request. Raw data and processed data will be made available at Gene Expression Omnibus (https://www.ncbi.nlm.nih.gov) upon acceptance of the manuscript for publication.
