# Supplementary material for: Molecular signature for senile and complicated cataracts derived from analysis of sumoylation enzymes and their substrates in human cataract lenses
Source: Aging Cell. 2020 Aug 22;19(10):e13222. doi: 10.1111/acel.13222 (PMC7576240; doi:10.1111/acel.13222)

Supplementary Data

1. EXPERIMENTAL PROCEDURES

1.1 Animals

The study was performed using 4 weeks old C57BL/6J mice, 4 weeks old Sprague-Dawley (SD) rats, and 3 months old pigs. Mice were housed in standard cages in a specific pathogen-free facility of Sun Yat-sen University. The room was maintained on a 12h light-dark cycle at a constant temperature of 25ºC and 50% humidity and the animals were fed with commercial laboratory food and sterilized water. SD rats were purchased from Zhongshan Medical School of Sun Yat-sen University. The 3 months old pigs were raised in the animal facility of Hunan Normal University. In all the cases, animal protocols to use the above species were approved by the IACUC of Zhongshan Ophthalmic Center of Sun Yat-sen University.

1.2 Collection of lens capsular epithelial samples

Collection of human capsular epithelia from cataract lenses of different age groups was approved by the Institutional Review Board of the Zhongshan Ophthalmic Center (ZOC). Informed consent was obtained from each of the cataract patients. For senile cataractous samples, the lens capsules from cataract patients were collected at surgery by the physicians in Zhongshan Ophthalmic Center of Sun Yat-sen University. According to the patient age, capsular samples from 50 to 59 years old were pooled together and labeled as 50s (Table S1); those from 60 to 69 years old were pooled together and labeled as 60s (Table S2); those from 70 to 79 years old were pooled together and labeled as 70s (Table S3), and those from 80 to 89 years old were pooled together and labeled as 80s

(Table S4). For complicated cataractous samples, patients with cataract and other complications including diabetes, glaucoma or other syndromes (Table S5) were pooled together for the study described in figure 5. As control, the capsular samples from the lenses of human donors (two female individuals with age of 45 and 56, and one male individual with age of 65), adult mice, rats and pigs as described in the above section were dissected out in the laboratory.

1.3 Lens organ culture

The 4 weeks old C57BL/6J mice were sacrificed by CO_2 inhalation. The eyeballs were removed and the lenses were carefully dissected by a posterior approach (Li et al., 1995, Li and Spector, 1996). Dissected lenses were placed in a 10-cm dish containing 20 ml meidium199, then incubated at 37°C with a 5% CO_2 gas phase for 12h. The medium 199 was prepared with ion-exchange double-distilled water and supplemented with 26mM $NaHCO_3$, with a pH adjusted to 7.2, then sterilized by filtration through 0.22um filter. After cultured for 12h, transparent lenses were selected for further experimentation.

1.4 Glucose oxidase (GO) treatment

For each sample, three transparent lenses were transferred into a 6-cm petri dish containing 7 ml of medium 199 supplemented with 10 mU glucose oxidase (GO) (Li et al., 1995; Gong et al., 2018), which continuously generates cytotoxic H_2O_2 in an average of 100 μM in a 24 h period.

1.5 Apoptosis assays

The percentage of apoptosis in GO-treated mouse lenses were determined by cellTiter-Glo® luminescent cell viability assay kit (G7573, Promega) (Crouch et al., 1993) and verified with live/dead viability/cytotoxicity kit (L3224, Thermofisher Scientific) according to the company instruction.

1.6 Total protein extraction and western blot analysis

Total proteins were extracted from cultured animal lenes with RIPA buffer (50 mM Tris·HCl (pH7.4), 150 mM NaCl, 2 mM EDTA, 1% NP-40, 0.1% SDS, 1% sodium deoxyholate) and homogenized as described below (Li et al., 1995; Li and Spector, 1996; Li et al, 2005; Yan et al., 200 and 2010; Gong et al., 2014 and 2018). For various capsular samples of lens epithelium, each pooled capsular samples from 23 to 41 patients (see Table S1 to S5 for details), or from 20 animal lenses were transferred to an Eppendorf tube containing 200 µl RIPA buffer and homogenized on ice with an Eppendorf tube micropestle (Brinkmann Instruments Inc.). For each sample, the protein concentration was determined as previously described (Li et al., 2006; Xiao et al., 2010). 100 µg of total proteins in each sample were separated by 8% SDS-PAGE gel and transferred into PVDF membranes. The protein blots were blocked with 5% non-fat milk in TBST (10 mM Tris HCl/pH8.0, 150 mM NaCl, 0.05% Tween-20) for 1 h at room temperature. Each membrane was then incubated with anti-Pax6 (rabbit monoclonal, 1:1000, CST, SAB4501345), anti-SUMO1 (rabbit polyclonal, 1:1000, CST, SAB1300037), SUMO2/3 (1:500; Enzo Life Sciences), anti-AOS1 (mouse monoclonal, 1:100, Santa Curz Biotechnology, sc376628), anti-UBA2 (rabbit polyclonal, 1:1000,

CST, 5293), anti-Ubc9 (rabbit monoclonal, 1:1000, CST, 4786), anti-PIAS1 (rabbit monoclonal, 1:1000, CST, 3550), anti-RanBP2 (mouse monoclonal, 1:500, Santa Curz Biotechnology, sc74518), anti-SENP1 (rabbit monoclonal, 1:1000, Sigma, SAB4501345), anti-SENP2 (rabbit polyclonal, 1:1000, Sigma, SAB1300037), anti-SENP3 (rabbit monoclonal, 1:1000, CST, 5591), anti-SENP5 (rabbit polyclonal, 1:1000, Proteintech, 19529-1-AP), anti-SENP6 (mouse monoclonal, 1:500, Santa Curz Biotechnology, sc-100585), anti-SENP7 (mouse monoclonal, 1:500, Santa Curz Biotechnology, sc-373821), anti-SENP8 (mouse monoclonal, 1:500, Santa Curz Biotechnology, sc-271498) and anti-β-Actin (mouse monoclonal, 1:1000, Beijing Ray Antibody Biotech, RM2001) at 4ºC overnight with mild shaking. After three 10 min washes with TBST, each blot was incubated with the HRP-conjugated secondary antibody (anti-mouse and rabbit IgG from CST) diluted at 1:2000 in blocking solution at room temperature for 1 h. The blots were visualized using a Tanon chemiluminescence system (China).

1.7 Automated western immunoblotting

The simple western immunoblots were performed on a PeggySue (ProteinSimple) as previously described (Dahl et al. 2016). Briefly, each sample was loaded with 0.9 μg total protein and then analyzed with the Size Separation Master Kit and Split Buffer (12-230 kDa) according to the manufacturer's standard instruction using the antibodies described above. The dilution factors are 1:100 for Pax6, Ubc9, Uba2, PIAS1, SENP3, SUMO2/3, and 1:50 for SENP1, SENP2, and SUMO1, as well as 1:20 for AOS1, RanBP2, SENP6, SENP7 and SENP8. The Campass software (Protein Simple, version

4.1.5) was used to program the PeggSue-robot and for presentation (and quantification) of the western blots. Output western blot style data were displayed with exposure time indicated, and the quantification data were displayed from the software-calculated average of seven exposures (1-512 s).

1.8 Protein solubility analysis

To compare protein solubility of human capsular epithelium samples from different age groups, total protein from each sample was first extracted as described above. The supernatant was considered as soluble protein. The remaining pellet was dissolved in modified protein extraction buffer with 5% CHAPs to replace 1% NP-40, and the suspended protein solutions were further sonicated on ice for 3 min, immersed in ice for 3 min. This cycle was repeated 5 times to help dissolving the pellet, the protein solution was then used for determination of protein concentration as described (Marshak et al. 1998). The protein solubility (Table S6) was calculated by dividing the initial soluble proteins with the total proteins from combination of two extractions.

1.9 References for Experimental Procedures

Crouch SP, Kozlowski R, Slater KJ and Fletcher J. (1993) The use of ATP bioluminescence as a measure of cell proliferation and cytotoxicity. J Immunol Methods. **160**(1):81-88.

Dimri GP, Lee X, Basile G, et al. A biomarker that identifies senescent human cells in culture and in aging skin *in vivo.* Proc Natl Acad Sci USA. 1995;**92**, 9363–9367.

Gong L, Fangyuan Liu, Zhen Xiong, Ruili Qi, Zhongwen Luo, Xiaodong Gong, Qian Nie, Qian Sun, Yunfei Liu, Wenjie Qing, Ling Wang, Lan Zhang, Xiangcheng Tang, Shan Huang, Ling Wang, Gen Li, Hong Ouyang, Mengqing Xiang, Quan Dong Nguyen,Yizhi Liu and David Wan-Cheng Li (2018) Heterochromatin protects retinal pigment epithelium cells from oxidative damage by silencing p53 target genes. Proc. Natl. Acad. Sci. USA **115,** E3987-E3995.

Gong L, Weike Ji, Xiao-Hui Hu,Wen-Feng Hu, Xiang-Cheng Tang, Zhao-Xia Huang, Ling Li, Mugen Liu, Shihua Xiang, Erxi Wu, Zachary Woodward, Yizhi Liu, Quan Dong Nguyen, and David Wan-Cheng Li (2014) Sumoylation differentially regulates Sp1 to control cell differentiation. Proc. Natl. Acad. Sci. USA **111,** 5574-5579.

Li DW, Liu JP, Mao YW, Xiang H, Wang J, Ma WY (2005) Calcium-activated RAF/MEK/ERK signaling pathway mediates p53-dependent apoptosis and is abrogated by alpha B-crystallin through inhibition of RAS activation. Mol Biol Cell 2005, **16,** 4437-4453.

Li DW, Liu JP, Schmid PC, Schlosser R, Feng H, Liu WB (2006) Protein serine/threonine phosphatase-1 dephosphorylates p53 at Ser-15 and Ser-37 to modulate its transcriptional and apoptotic activities. Oncogene **25**, 3006-3022.

Li WC, Kuszak JR, Dunn K, Wang RR, Ma WC, Wang GM, Spector A, Leib M, Cotliar AM, Weiss M, Espy J, Howard G, Farris RL, Auran J, Donn A, Hofeldt A, Mackay C, Merriam J, Mittl R, Smith TR. (1995). Lens epithelial cell apoptosis appears to be a common cellular basis for non-congenital cataract development in humans and animals. J. Cell Biol. **130**, 169–181.

Li WC, Spector A. (1996). Lens Eepithelial cell apoptosis is an early event in the development of UVB-induced cataract. Free Radic. Biol. Med**. 20**, 301–311.

Marshak DR, Kadonaka JT, Burgess RR, Knuth MW, Brennan WA, Lin SH. (1996). Strategies for protein purification and characterization. A laboratory course manual. Cold Spring Harbor Laboratory Press.

Xiao L, Gong LL, Yuan D, Deng M, Zeng XM, Chen LL (2010) Protein phosphatase-1 regulates Akt1 signal transduction pathway to control gene expression, cell survival and differentiation. Cell Death Differ. **17,** 1448-1462.

Yan Q, Gong L, Deng M, Zhang L, Sun S, Liu J, Ma H, Yuan D, Chen PC, Hu X, Liu J, Qin J, Xiao L, Huang XQ, Zhang J, Li DW. (2010). Sumoylation activates the transcriptional activity of Pax-6, an important transcription factor for eye and brain development. Proc. Natl. Acad. Sci. USA **107**, 21034-9.

Yan Q, Liu JP, Li DW (2006) Apoptosis in lens development and pathology. Differentiation. **74,** 195-211.

Yan Q, Liu WB, Qin J, Liu J, Chen HG (2007) Protein phosphatase-1 modulates the function of Pax-6, a transcription factor controlling brain and eye development. J. Biol. Chem. 282, 13954-13965.

2. SUPPLEMENTARY TABLES

Table S1. Senile cataract patients' information (50-59 years old)

Number	Gender	Age	Diagnosed subtype
P1	**Female**	**50**	**Nuclear Cataract**
P2	**Female**	**50**	**Cortical Cataract**
P3	**Female**	**51**	**Cortical Cataract**
P4	**Female**	**51**	**Nuclear Cataract**
P5	**Female**	**51**	**Cortical Cataract**
P6	**Female**	**51**	**Cortical Cataract**
P7	**Male**	**51**	**Cortical Cataract**
P8	**Male**	**52**	**Cortical Cataract**
P9	**Male**	**52**	**Cortical Cataract**
P10	**Male**	**52**	**Nuclear Cataract**
P11	**Male**	**53**	**Cortical Cataract**
P12	**Female**	**53**	**Nuclear Cataract**
P13	**Female**	**55**	**Nuclear Cataract**
P14	**Female**	**55**	**Cortical Cataract**
P15	**Female**	**56**	**Cortical Cataract**
P16	**Female**	**56**	**Cortical Cataract**
P17	**Female**	**56**	**Cortical Cataract**
P18	**Male**	**56**	**Nuclear Cataract**
P19	**Male**	**57**	**Nuclear Cataract**
P20	**Female**	**57**	**Nuclear Cataract**
P21	**Female**	**57**	**Cortical Cataract**
P22	**Female**	**57**	**Cortical Cataract**
P23	**Male**	**58**	**Cortical Cataract**
P24	**Female**	**58**	**Nuclear Cataract**
P25	**Male**	**58**	**Cortical Cataract**
P26	**Female**	**59**	**Cortical Cataract**
P27	**Female**	**59**	**Cortical Cataract**
P28	**Female**	**57**	**Nuclear Cataract**
P29	**Female**	**58**	**Cortical Cataract**
P30	**Male**	**58**	**Nuclear Cataract**
P31	**Male**	**59**	**Nuclear Cataract**

Samples displayed in red were used for automated western blot analysis.

Table S2. Senile cataract patients' information (60-69 years old)

Number	Gender	Age	Diagnosed subtype
P1	Female	60	Cortical Cataract
P2	Female	60	Cortical Cataract
P3	Male	60	Nuclear Cataract
P4	Female	61	Cortical Cataract
P5	Female	62	Nuclear Cataract
P6	Male	62	Cortical Cataract
P7	Male	62	Cortical Cataract
P8	Female	63	Nuclear Cataract
P9	Female	63	Cortical Cataract
P10	Female	63	Cortical Cataract
P11	Female	63	Cortical Cataract
P12	Male	63	Nuclear Cataract
P13	Female	64	Cortical Cataract
P14	Male	64	Nuclear Cataract
P15	Male	65	Nuclear Cataract
P16	Female	65	Cortical Cataract
P17	Female	65	Cortical Cataract
P18	Female	65	Nuclear Cataract
P19	Female	65	Cortical Cataract
P20	Male	65	Nuclear Cataract
P21	Female	66	Cortical Cataract
P22	Male	66	Cortical Cataract
P23	Female	66	Cortical Cataract
P24	Male	66	Cortical Cataract
P25	Female	66	Nuclear Cataract
P26	Female	66	Cortical Cataract
P27	Female	66	Cortical Cataract
P28	Female	67	Cortical Cataract
P29	Female	67	Nuclear Cataract
P30	Female	67	Nuclear Cataract
P31	Female	67	Cortical Cataract
P32	Female	68	Cortical Cataract
P33	Male	68	Nuclear Cataract
P34	Male	69	Nuclear Cataract
P35	Female	67	Nuclear Cataract
P36	Female	67	Nuclear Cataract
P37	Male	67	Posterior Subcapsular Cataract
P38	Male	68	Nuclear Cataract

Samples displayed in red were used for automated western blot analysis.

Table S3. Senile cataract patients' information (70-79 years old)

Number	Gender	Age	Diagnosed subtype
P1	Female	70	Cortical Cataract
P2	Female	70	Cortical Cataract
P3	Female	71	Cortical Cataract
P4	Male	71	Nuclear Cataract
P5	Male	71	Nuclear Cataract
P6	Female	72	Cortical Cataract
P7	Female	72	Cortical Cataract
P8	Male	72	Cortical Cataract
P9	Female	73	Cortical Cataract
P10	Male	73	Cortical Cataract
P11	Female	73	Cortical Cataract
P12	Female	73	Nuclear Cataract
P13	Male	73	Cortical Cataract
P14	Female	73	Cortical Cataract
P15	Male	74	Nuclear Cataract
P16	Female	74	Nuclear Cataract
P17	Male	74	Nuclear Cataract
P18	Male	74	Cortical Cataract
P19	Female	75	Nuclear Cataract
P20	Female	75	Cortical Cataract
P21	Female	75	Nuclear Cataract
P22	Male	75	Nuclear Cataract
P23	Female	76	Cortical Cataract
P24	Male	76	Nuclear Cataract
P25	Male	76	Nuclear Cataract
P26	Female	76	Cortical Cataract
P27	Female	77	Cortical Cataract
P28	Female	77	Nuclear Cataract
P29	Female	78	Nuclear Cataract
P30	Female	78	Cortical Cataract
P31	Male	78	Cortical Cataract
P32	Female	79	Cortical Cataract
P33	Male	79	Cortical Cataract
P34	Male	75	Nuclear Cataract
P35	Male	79	Nuclear Cataract
P36	Female	79	Nuclear Cataract
P37	Female	79	Nuclear Cataract

Samples displayed in red were used for automated western blot analysis.

Table S4. Senile cataract patients' information (80-90 years old)

Number	Gender	Age	Diagnosed subtype
P1	Male	80	Nuclear Cataract
P2	Female	80	Nuclear Cataract
P3	Female	81	Cortical Cataract
P4	Male	81	Nuclear Cataract
P5	Male	81	Cortical Cataract
P6	Male	81	Nuclear Cataract
P7	Female	82	Cortical Cataract
P8	Female	82	Nuclear Cataract
P9	Male	82	Nuclear Cataract
P10	Female	83	Cortical Cataract
P11	Male	83	Cortical Cataract
P12	Male	84	Cortical Cataract
P13	Female	84	Cortical Cataract
P14	Female	86	Cortical Cataract
P15	Female	86	Cortical Cataract
P16	Male	87	Cortical Cataract
P17	Male	88	Nuclear Cataract
P18	Female	88	Cortical Cataract
P19	Female	89	Nuclear Cataract
P20	Female	89	Nuclear Cataract
P21	Female	89	Cortical Cataract
P22	Female	89	Nuclear Cataract
P23	Male	90	Cortical Cataract
P24	Male	83	Nuclear Cataract
P25	Male	85	Nuclear Cataract
P26	Female	83	Cortical Cataract
P27	Female	84	Nuclear Cataract

Samples displayed in red were used for automated western blot analysis.

Table S5. Complicated cataract patients' information

Number	Gender	Age	Complication
P1	Male	39	High Myopia
P2	Female	48	Diabetes
P3	Female	50	Glaucoma
P4	Male	51	High Myopia
P5	Female	52	High Myopia
P6	Female	54	Glaucoma
P7	Female	55	High Myopia
P8	Male	56	Retinal Detachment
P9	Female	57	Glaucoma
P10	Female	57	Diabetes
P11	Male	59	Diabetes
P12	Female	60	Retinal Detachment
P13	Male	61	Uveitis
P14	Female	61	Retinal Detachment
P15	Female	61	Retinitis Pigmentosa
P16	Male	63	Glaucoma
P17	Male	63	Uveitis
P18	Male	64	Retinitis Pigmentosa
P19	Female	64	Retinitis Pigmentosa
P20	Female	66	Diabetes
P21	Male	67	Glaucoma
P22	Male	70	Retinal Detachment
P23	Female	71	Glaucoma
P24	Male	72	Retinal Detachment
P25	Male	72	Glaucoma
P26	Female	72	Retinal Detachment
P27	Male	74	High Myopia
P28	Male	75	Uveitis
P29	Female	76	Diabetes
P30	Female	77	Diabetes
P31	Female	78	Retinitis Pigmentosa
P32	Female	79	Retinal Detachment
P33	Male	79	Glaucoma
P34	Female	79	Glaucoma
P35	Female	80	Diabetes
P36	Male	80	Glaucoma
P37	Male	81	Retinitis Pigmentosa
P38	Male	81	Glaucoma
P39	Male	85	Glaucoma
P40	Male	89	Glaucoma
P41	Male	90	Retinal Detachment
P42	Female	23	Uveitis

P43	**Female**	**29**	**Retinal Detachment**
P44	**Male**	**34**	**Retinitis Pigmentosa**
P44	**Male**	**38**	**High Myopia**
P45	**Male**	**38**	**Glaucoma**
P46	**Male**	**43**	**High Myopia**
P47	**Male**	**44**	**Hypertension**
P48	**Female**	**45**	**Retinal Detachment**
P49	**Male**	**47**	**Uveitis**
P50	**Male**	**48**	**Glaucoma**
P51	**Female**	**49**	**High Myopia**
P52	**Male**	**50**	**Retinitis Pigmentosa**
P53	**Female**	**53**	**Retinal Detachment**
P54	**Female**	**53**	**Diabetes**
P55	**Male**	**62**	**Hypertension**
P56	**Male**	**63**	**Retinal Detachment**
P57	**Female**	**64**	**Diabetes**

Samples displayed in red were used for automated western blot analysis.

Table S6. Result of the soluble proteins in different age group samples

Age groups	Total protein (μg)	Soluble protein (μg)	insoluble protein (μg)	The proportion of soluble protein
50s	287.12	254.17	32.95	100%
60s	215.73	188.27	27.46	87%
70s	207.89	180.43	27.46	87%
80s	211.02	178.08	32.94	84%

3. LEGENDS TO SUPPLEMENTARY FIGURES

FIGURE S1. Automated western blot analysis of ligase 1 subunits, AOS1 and UBA2 in normal and cataractous human lenses of different age groups. The automated western immunoblots were performed on a PeggySue (ProteinSimple) as described recently (Dahl et al. 2016). Briefly, each sample was loaded with 0.9 μg total protein and then analyzed with the Size Separation Master Kit and Split Buffer (12-230 kDa) according to the manufacturer's standard instruction using anti-AOS1 and anti-UBA2 antibodies (for antibody information, see Experimental Procedures) with a dilution factor of 1:20 and 1:100, respectively. The Campass software (Protein Simple, version 4.1.5) was used to program the PeggSue-robot and for presentation (**A**) and quantification (B-E). Output western blot style data (**A**) were displayed with exposure time indicated, and the quantification data (B-E) were displayed from the software-calculated average of seven exposures (1-512 s). **A**. Besides AOS1 and UBA2 samples, β-actin with 1 second exposure was included as loading control. **B** and **C**. Quantification results show gender difference. Each bar represents an average of two samples for cataract lenses but one sample for normal human lens. **D** and **E**. Quantification results show age difference.

FIGURE S2. Automated western blot analysis of ligase 2, UBC9 in normal and cataractous human lenses of different age groups. The automated western immunoblots were performed on a PeggySue (ProteinSimple) as described recently (Dahl et al. 2016). Briefly, each sample was loaded with 0.9 μg total protein and then analyzed with the Size Separation Master Kit and Split Buffer (12-230 kDa) according to the manufacturer's standard instruction using anti-UBC9 antibody (for antibody information,

see Experimental Procedures) with a dilution factor of 1:100. The Campass software (Protein Simple, version 4.1.5) was used to program the PeggSue-robot and for presentation (**A**) and quantification (B-C). Output western blot style data (A) were displayed with exposure time indicated, and the quantification data (B-C) were displayed from the software-calculated average of seven exposures (1-512 s). B. Quantification results show gender difference. Each bar represents an average of two samples for cataract lenses but one sample for normal human lens. **C**. Quantification results show age difference.

FIGURE S3. Automated western blot analysis of ligase 3, PIAS1 in normal and cataractous human lenses of different age groups. The automated western immunoblots were performed on a PeggySue (ProteinSimple) as described recently (Dahl et al. 2016). Briefly, each sample was loaded with 0.9 μg total protein and then analyzed with the Size Separation Master Kit and Split Buffer (12-230 kDa) according to the manufacturer's standard instruction using anti-PIAS1 antibody (for antibody information, see Experimental Procedures) with a dilution factor of 1:100. The Campass software (Protein Simple, version 4.1.5) was used to program the PeggSue-robot and for presentation (**A**) and quantification (B-C). Output western blot style data (A) were displayed with exposure time indicated, and the quantification data (B-C) were displayed from the software-calculated average of seven exposures (1-512 s). B. Quantification results show gender difference. Each bar represents an average of two samples for cataract lenses but one sample for normal human lens. **C**. Quantification results show age difference.

FIGURE S4. Automated western blot analysis of desumoylation enzyme, SENP3 in normal and cataractous human lenses of different age groups. The automated western immunoblots were performed on a PeggySue (ProteinSimple) as described recently (Dahl et al. 2016). Briefly, each sample was loaded with 0.9 μg total protein and then analyzed with the Size Separation Master Kit and Split Buffer (12-230 kDa) according to the manufacturer's standard instruction using anti-SENP3 antibody (for antibody information, see Experimental Procedures) with a dilution factor of 1:100. T The Campass software (Protein Simple, version 4.1.5) was used to program the PeggSue-robot and for presentation (A) and quantification (B-C). Output western blot style data (A) were displayed with exposure time indicated, and the quantification data (B-C) were displayed from the software-calculated average of seven exposures (1-512 s). B. Quantification results show gender difference. Each bar represents an average of two samples for cataract lenses but one sample for normal human lens. C. Quantification results show age difference.

FIGURE S5. Automated western blot analysis of desumoylation enzyme, SENP7 in normal and cataractous human lenses of different age groups. The automated western immunoblots were performed on a PeggySue (ProteinSimple) as described recently (Dahl et al. 2016). Briefly, each sample was loaded with 0.9 μg total protein and then analyzed with the Size Separation Master Kit and Split Buffer (12-230 kDa) according to the manufacturer's standard instruction using anti-SENP7 antibody (for antibody information, see Experimental Procedures) with a dilution factor of 1:20. The

Campass software (Protein Simple, version 4.1.5) was used to program the PeggSue-robot and for presentation (**A**) and quantification (B-C). Output western blot style data (A) were displayed with exposure time indicated, and the quantification data (B-C) were displayed from the software-calculated average of seven exposures (1-512 s). B. Quantification results show gender difference. Each bar represents an average of two samples for cataract lenses but one sample for normal human lens. **C**. Quantification results show age difference.

FIGURE S6. Automated western blot analysis of desumoylation enzyme, SENP8 in normal and cataractous human lenses of different age groups. The automated western immunoblots were performed on a PeggySue (ProteinSimple) as described recently (Dahl et al. 2016). Briefly, each sample was loaded with 0.9 μg total protein and then analyzed with the Size Separation Master Kit and Split Buffer (12-230 kDa) according to the manufacturer's standard instruction using anti-SENP8 antibody (for antibody information, see Experimental Procedures) with a dilution factor of 1:20. The Campass software (Protein Simple, version 4.1.5) was used to program the PeggSue-robot and for presentation (**A**) and quantification (B-C). Output western blot style data (A) were displayed with exposure time indicated, and the quantification data (B-C) were displayed from the software-calculated average of seven exposures (1-512 s). B. Quantification results show gender difference. Each bar represents an average of two samples for cataract lenses but one sample for normal human lens. **C**. Quantification results show age difference.

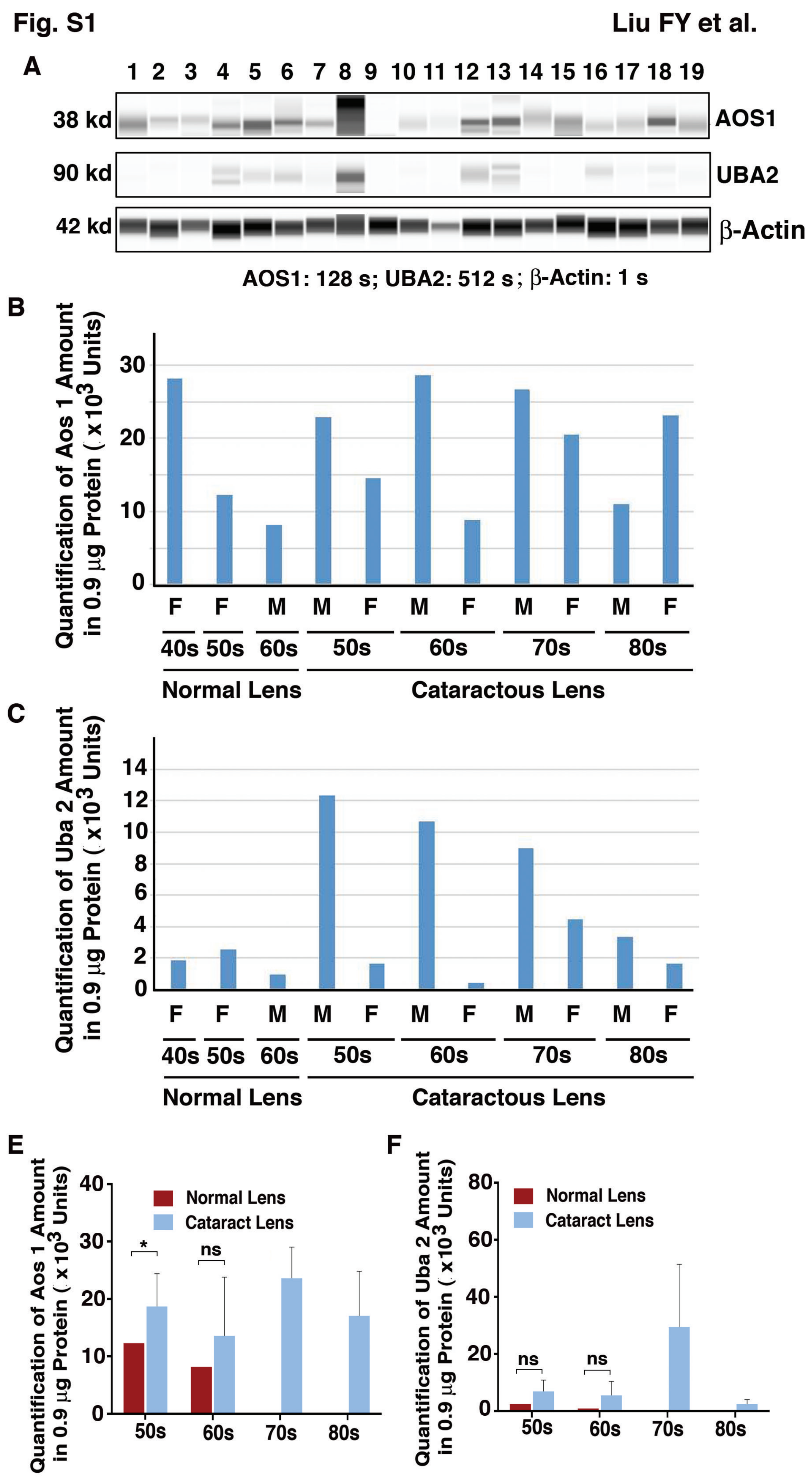
Fig. S1
Liu FY et al.
A
1 2 3 4 5 6 7 8 9 10 11 12 13 14 15 16 17 18 19
38 kd
AOS1
90 kd
UBA2
42 kd
β-Actin
AOS1: 128 s; UBA2: 512 s; β-Actin: 1 s
B
Quantification of Aos 1 Amount in 0.9 μg Protein (x10^3 Units)
F F M M F M F M F M F
40s 50s 60s 50s 60s 70s 80s
Normal Lens
Cataractous Lens
C
Quantification of Uba 2 Amount in 0.9 μg Protein (x10^3 Units)
F F M M F M F M F M F
40s 50s 60s 50s 60s 70s 80s
Normal Lens
Cataractous Lens
E
Quantification of Aos 1 Amount in 0.9 μg Protein (x10^3 Units)
Normal Lens
Cataract Lens
*
ns
50s 60s 70s 80s
F
Quantification of Uba 2 Amount in 0.9 μg Protein (x10^3 Units)
Normal Lens
Cataract Lens
ns
ns
50s 60s 70s 80s

Fig. S2 Liu et al.

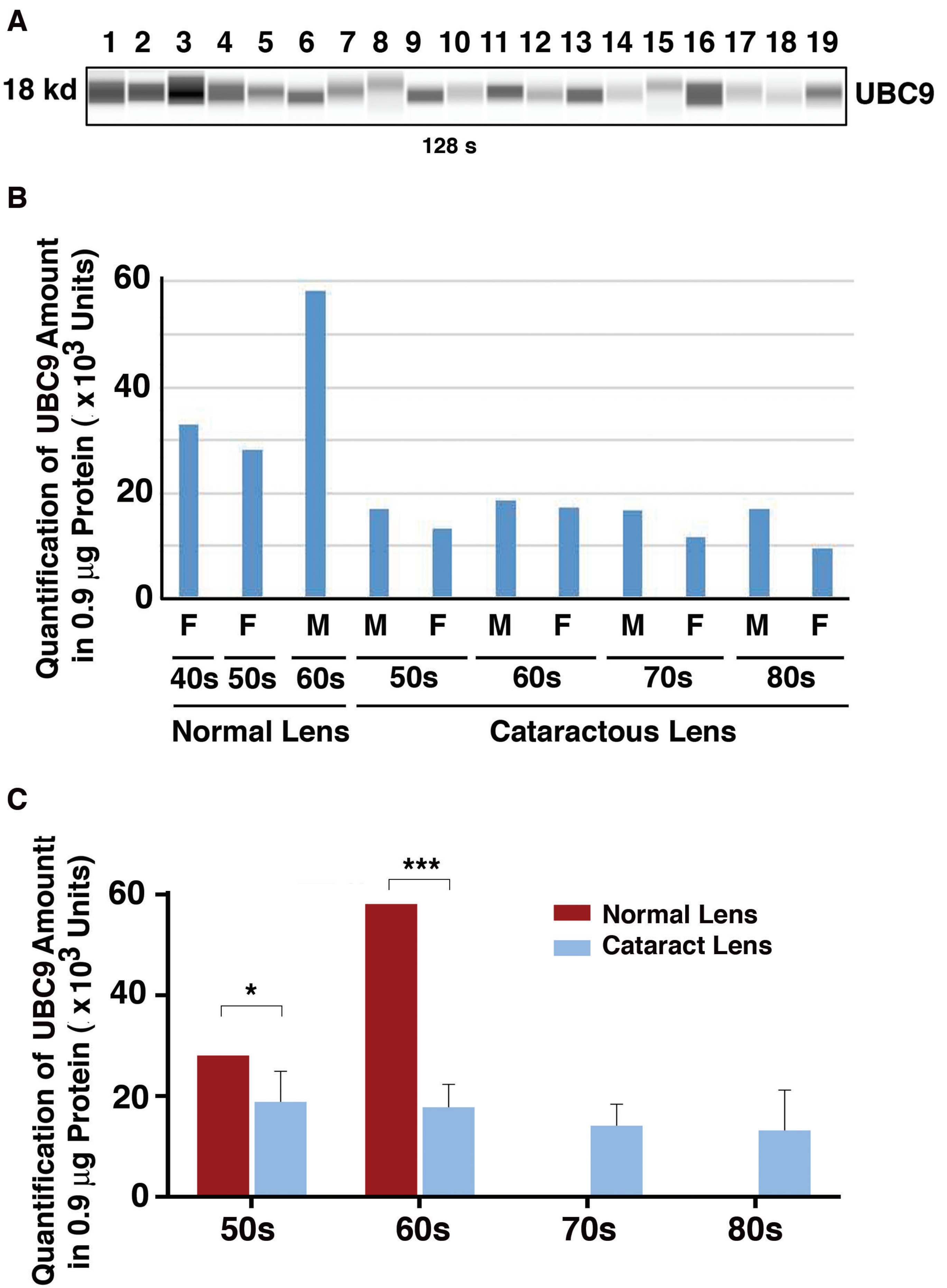

Fig. S3 Liu et al.

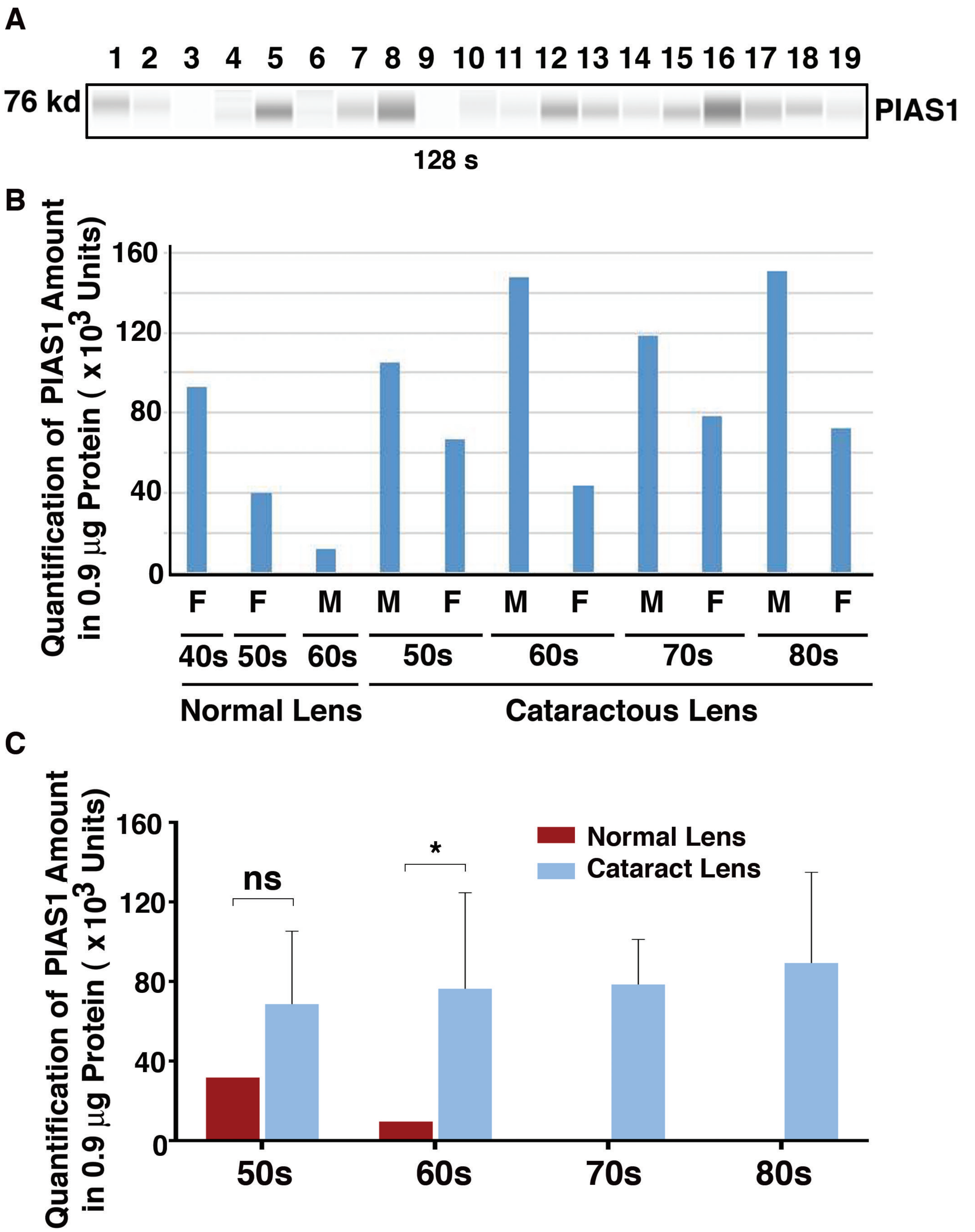

Fig. S4 **Liu et al.**

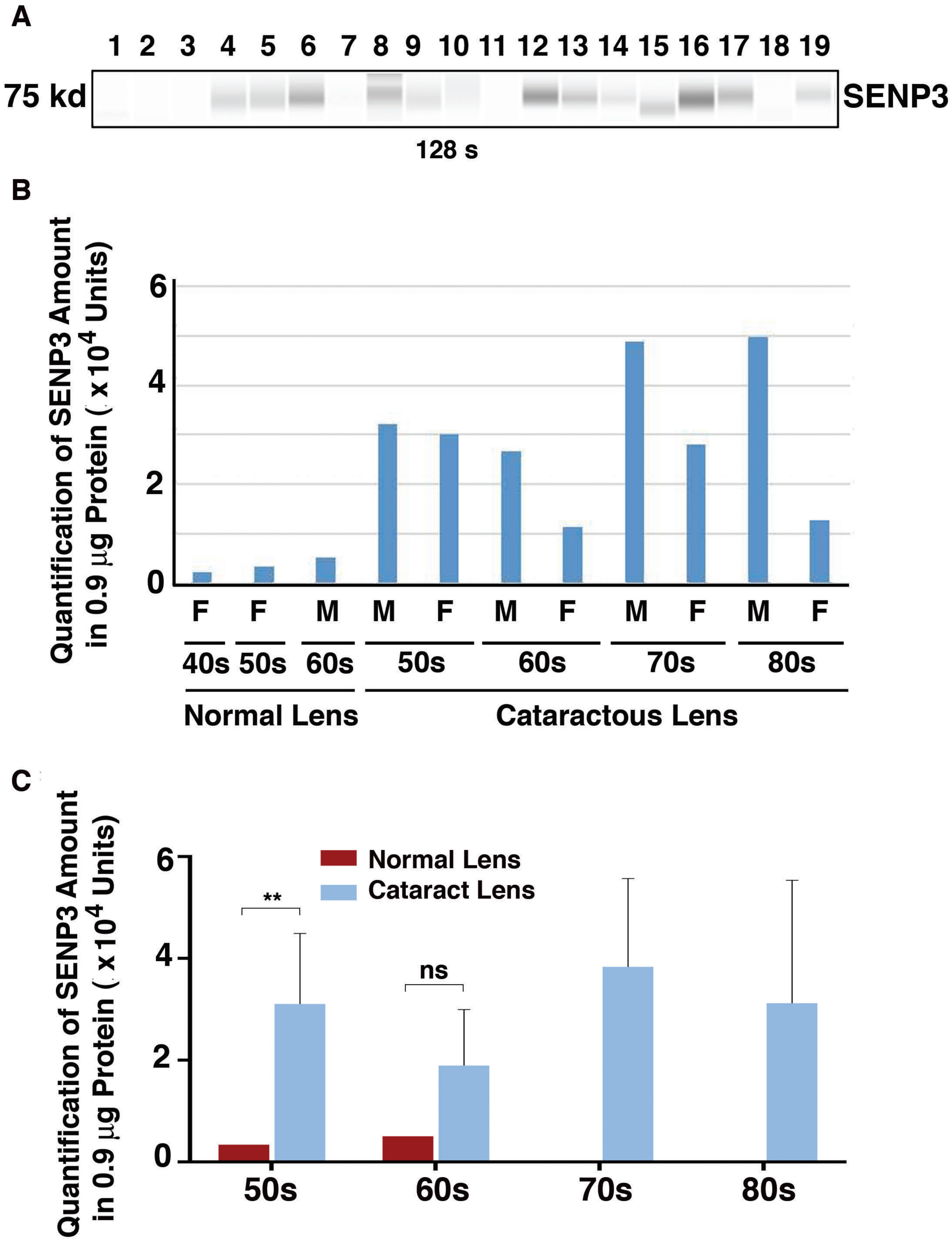

Fig. S5 **Liu et al.**

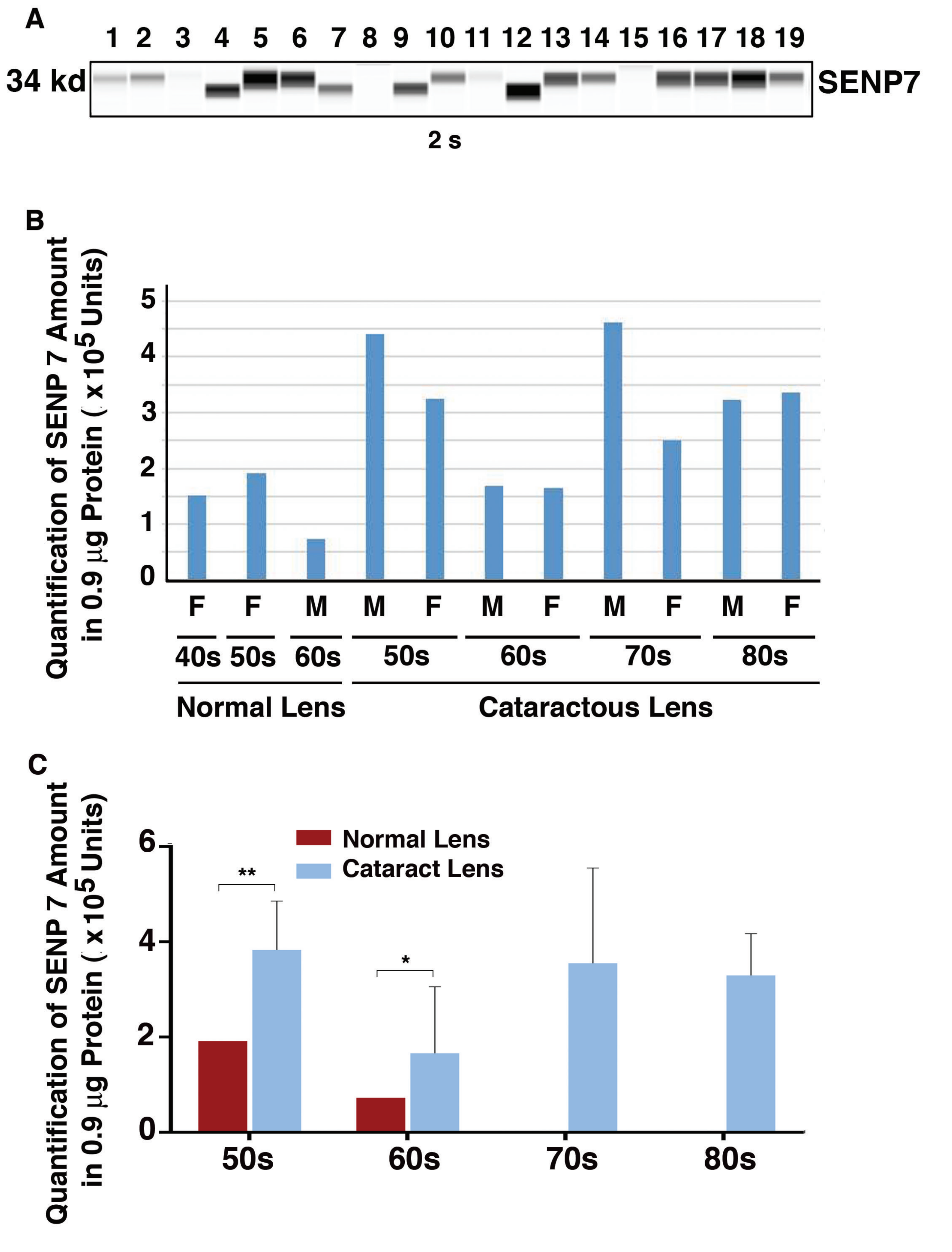

Fig. S6

Liu et al.

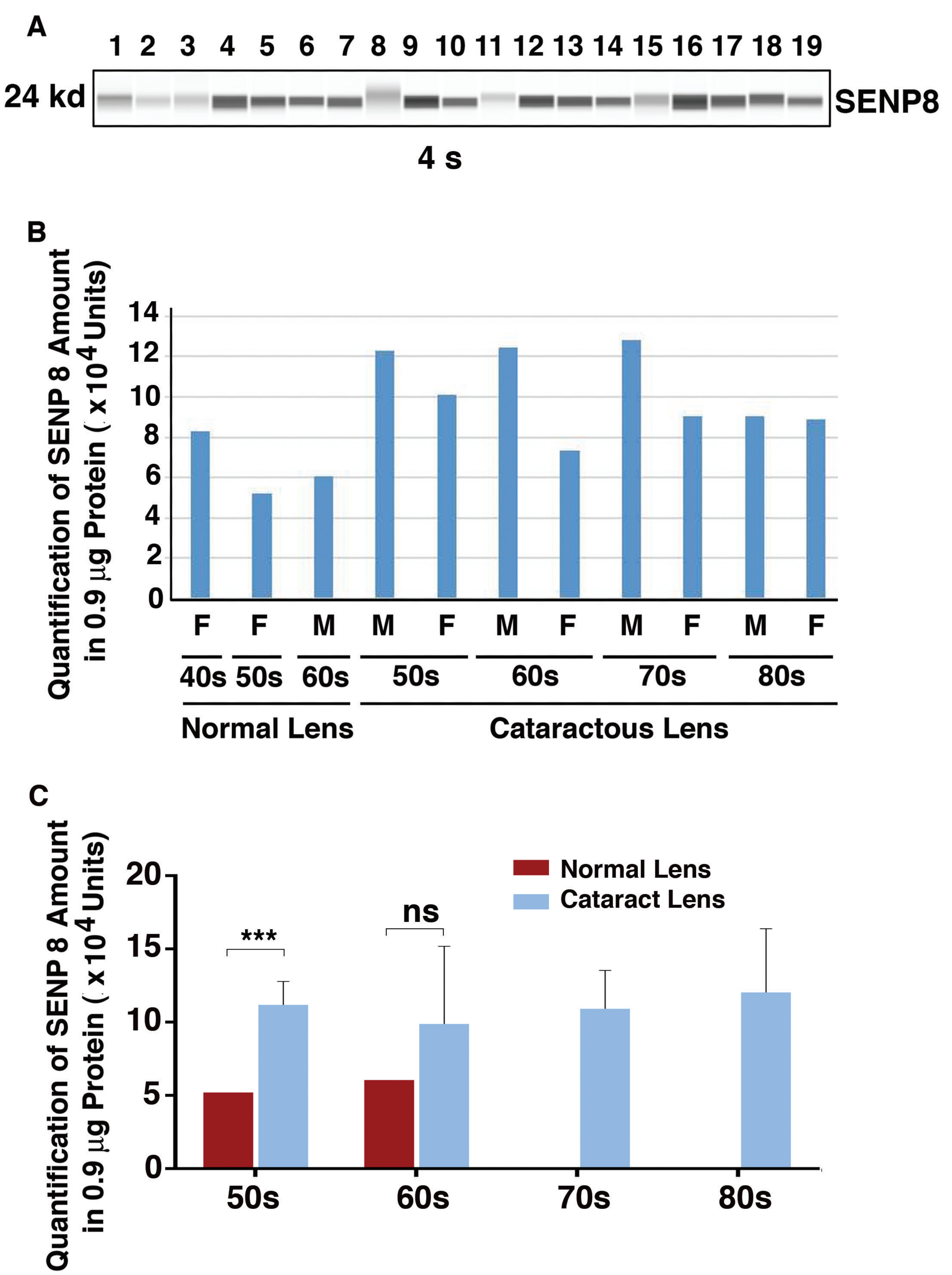

Supplement: Supplementary file 1 — Supplementary Material [file ACEL-19-e13222-s001.pdf]
